# Lipid Biomarker Research in Bipolar Disorder: A Scoping Review of Trends, Challenges, and Future Directions

**DOI:** 10.1016/j.bpsgos.2023.07.004

**Published:** 2023-07-23

**Authors:** John Kim Hiller, Andreas Jangmo, Martin Steen Tesli, Piotr Pawel Jaholkowski, Eva Zsuzsanna Hoseth, Nils Eiel Steen, Marit Haram

**Affiliations:** a Faculty of Medicine, University of Oslo, Oslo, Norway; b Department of Mental Disorders, Norwegian Institute of Public Health, Oslo, Norway; c Department of Medical Epidemiology and Biostatistics, Karolinska Institute, Stockholm, Sweden; d Centre for Research and Education in Forensic Psychiatry, Department of Mental Health and Addiction, Oslo University Hospital, Oslo, Norway; e Norwegian Centre for Mental Disorders Research, Division of Mental Health and Addiction, Oslo University Hospital, Oslo, Norway; f Institute of Clinical Medicine, University of Oslo, Oslo, Norway; g Clinic of Mental Health and Addiction, Møre and Romsdal Health Trust, Kristiansund, Norway; h Division of Mental Health and Addiction, Oslo University Hospital, Oslo, Norway

**Keywords:** Biomarker, Bipolar disorder, Cholesterol, Lipid, Lipidomics, Scoping review

## Abstract

Bipolar disorder (BD) is a disabling disorder with heterogeneous symptom profiles and trajectories. Like many other neuropsychiatric disorders, clinical decision making related to diagnoses and choice of treatment is based on clinical assessments alone, and risk prediction for treatment success or resistance at an individual level remains sparse. An enormous effort to add biological markers to this risk prediction is ongoing. The role of lipids in normal brain functioning is well established, and several hypotheses about the role of lipids in the pathogenesis of neuropsychiatric disorders, including BD, have been made. The frequent comorbidity between neuropsychiatric disorders and cardiovascular disease, the genetic overlap of risk genes for severe mental disorders and genes involved in lipid regulation, and the lipid-altering effects of antipsychotics and mood stabilizers indicate that lipids could hold promise as biomarkers for neuropsychiatric disorders, including BD. To date, reviews of lipid biomarkers in schizophrenia and major depression have noted caveats for future investigations, while reviews of lipid biomarker research in BD is missing. In the current scoping review, we present a comprehensive overview of trends in previous research on lipid biomarkers in BD. The current literature varies greatly in the phenotypes investigated and study designs, leading to divergent findings. Small sample size; potential confounders related to physical activity, nutritional status, and medication use; and cross-sectional designs were frequently reported limitations. Future research may benefit from pivoting toward utilization of newer laboratory techniques such as lipidomics, but consistent use of study methods across cohorts is also needed.

Bipolar disorder (BD) is a severe mental disorder that often leads to lifelong impairment in function and cognition, as well as increased mortality and high rates of comorbidity (1, 2, 3, 4). The global lifetime prevalence of BD is estimated to be 2% to 3% (5,6), and in most cases, the illness begins during adolescence or early adulthood (7,8). BD has a high degree of heterogeneity, characterized by fluctuating mood and energy states with episodes of mania, hypomania, and depression of varying severity and can also include psychotic features such as delusions and hallucinations (1,9). BD has a high rate of heritability (10,11), and family history is recognized as an important risk factor for developing the disorder (8,12).

Lipids and lipid metabolism may play key roles in the pathophysiology of neuropsychiatric disorders, including BD. This is due to the great abundance of lipid species in the human brain, the close relationship between lipid metabolism and cardiovascular disease, and the lipid-altering effects of medication used to treat such disorders (13, 14, 15, 16, 17). Notably, studies indicate that dysregulation of phospholipid metabolism and lipid peroxidation due to oxidative stress are important (13,15,18, 19, 20). Moreover, there is a strong association between neuropsychiatric disorders and the development of dyslipidemia and cardiovascular disease (20, 21, 22, 23, 24), as well as other inflammatory conditions with alterations in lipid metabolic pathways involved in the immune system, such as arachidonic acid metabolism (14,25,26). Furthermore, recent genome-wide association studies have uncovered overlap between susceptibility loci for neuropsychiatric disorders like schizophrenia and BD and genes linked to the regulation of lipid levels (27, 28, 29). In addition, it has been suggested that antilipidemic medications such as statins aid in the treatment of depressive symptoms (30, 31, 32, 33). These findings suggest that lipids and biomolecules involved in lipid metabolism may represent promising potential biomarkers in the field of neuropsychiatric disorders.

Given the critical roles that lipids play in neurodevelopment and the normal functioning brain, molecular markers may significantly aid in the prediction and detection of neuropsychiatric disorders and their prognoses (1,6,34, 35, 36). Unfortunately, progress in the search for diagnostic and prognostic biomarkers in psychiatry is somewhat slower than in other medical fields (37,38), and there are currently no definitive molecular biomarkers of pathophysiological processes associated with such disorders (9,35,38, 39, 40). Nevertheless, studies have identified multiple potential candidates for molecular markers (35,38, 39, 40, 41, 42, 43), and molecules related to lipid metabolism are emerging as interesting biomarkers (39,41, 42, 43, 44, 45, 46). Such markers may offer prospects in precision medicine through new modalities of early detection of disease, diagnostics, and prognostics.

Previous reviews have focused on lipid markers in other severe mental illnesses such as schizophrenia (45, 46, 47, 48) and major depression (49, 50, 51). Despite promising results, these fields of research remain mainly inconclusive. To the best of our knowledge, no reviews on lipid markers in BD exist. Given the nature of scoping reviews, we included studies with a wide range of designs and previous research in lipid biomarker research since 1990 in individuals with BD. With the main objective of providing a comprehensive overview of existing literature, we sought to identify knowledge gaps and provide guidance for future research on this topic. In particular, we aimed to focus on trends in the number of studies performed and case sample sizes investigated. We aimed to map methods used for investigations in terms of laboratory methods used over time, selection of lipid parameters and phenotypes, and study limitations reported. In addition, we aimed to present key study findings.

## Methods

The current scoping review was conducted in accordance with the Preferred Reporting Items for Systematic Reviews and Meta-Analyses (PRISMA)–extension for Scoping Reviews checklist and guidelines for scoping reviews (52). The methodological framework and study structure applied in the paper was adapted from Arksey and O’Malley’s scoping review methodology literature (53), as well as the more recently published guidelines provided by the Joanna Briggs Institute of the University of Adelaide, Australia (54). A protocol for this scoping review was registered on the Open Science Framework website (55), following the PRISMA-protocols guidelines for review protocols (56). Some alterations were applied to the review protocol after it was registered, as summarized in Supplement Note S1.

### Search Strategy

A comprehensive literature search was conducted by an experienced academic librarian in the electronic bibliographic databases MEDLINE (Ovid), Embase (Ovid), APA PsycInfo (Ovid), and Scopus on September 9, 2022. The search consisted of a combination of terms relevant to the role of lipids in BD. Documentation and extensive details of searches are reported in Supplement Note S2.

### Eligibility Criteria

#### Population

All individuals diagnosed with BD according to any ICD or DSM classification system editions.

#### Concept

Measurement of lipid levels in body fluids of patients as potential biomarkers.

#### Context

Any kind of lipid species measured in any phase of any BD type.

#### Exclusion Criteria

• Non-English language papers.
• Papers published before 1990.
• Letters, editorials, comments, conference abstracts, case reports, and unpublished literature.
• Animal and in vitro studies.
• Studies in postmortem tissue and tissues that are not body fluids (e.g., brain tissue, biopsies, in vivo tissue spectroscopies) because these are not applicable in clinical settings.
• Studies that only measured molecules related to lipids, but are not lipids themselves, including molecules related to lipid peroxidation.
• Studies that measured dysregulated lipid levels specifically as a result of medication.
• Epidemiological studies that did not investigate associations between lipids and clinical correlates in BD.
• Reports that assessed metabolic syndrome in BD without distinguishing between individual metabolic syndrome lipid parameters.
• Reports that investigated associations between lipids and BD for reasons other than assessing lipids as potential biomarkers for BD, such as explaining the prevalence of metabolic syndrome in BD.

### Selection of Sources of Evidence

The selection of reports was conducted in accordance with the PRISMA 2020 statement (57). With the use of Rayyan (58), the study selection process consisted of 2 steps. Firstly, 2 reviewers (JKH and MH) independently screened the records against eligibility criteria based on titles and abstracts. Any disagreements were solved by discussion and consensus between the 2 researchers. Secondly, the records included from the first round of screening were sought for retrieval and screened in full text, and reasons for exclusion were registered for every dismissed report (Table S1). As in the first step, disagreements were resolved by discussion and consensus between the researchers.

### Data Charting and Analysis

The main reviewer (JKH) performed the data extraction with items decided a priori, using a standard extraction form. Quality assurance was performed by MH. The data were charted using Microsoft Excel (Microsoft Corporation). Data items that were extracted included information about the publication (title, author, year), as well as study design, study aims, lipids and phenotypes investigated, relevant findings, and reported limitations. All data items are listed and described in Table S2.

Some simplifications were made during data charting. The study designs were charted by the study author’s reported study design, without performing further evaluation. We also did not chart data on findings that were not related to the lipids investigated or the relevant phenotypes. Regarding the findings in case-control studies between BD and other psychiatric disorders, negative results and results that were determined to be irrelevant were not charted. Similarly, under “Authors’ interpretation of outcomes,” we did not chart data on interpretations that were not directly relevant to the charted findings.

Number of reports, number of cases included in studies over time, distribution of laboratory methods used, lipid parameters, and phenotypes investigated were selected for bar plots using the R package ggplot2 (59). The number of cases was based on the number of subjects with BD. Some reports utilized more than one laboratory method, and in these instances, all laboratory methods were charted, but for simplicity, only the most advanced method was presented in a figure. Similarly, some reports investigated several lipids and phenotypes, which are reflected in the bar plots. Reported study limitations were counted in a table and presented, based on the data extraction. Main study results and contradictory findings were reported in a descriptive manner, without performing a critical appraisal or full synthesis of the results.

## Results

### Selection of Sources of Evidence

**Figure 1:**
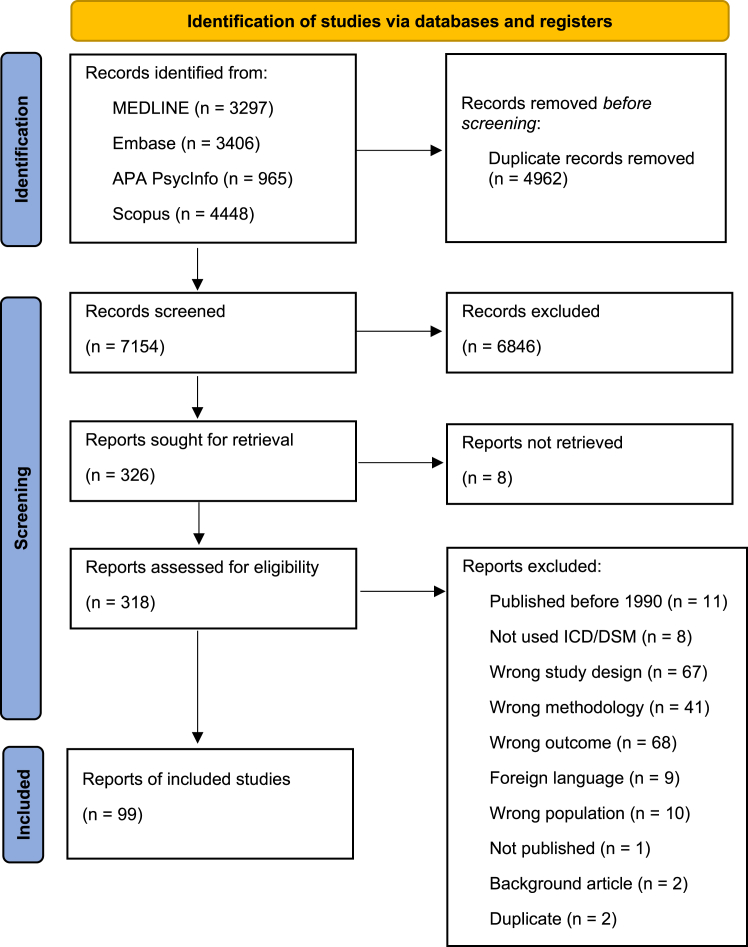
PRISMA (Preferred Reporting Items for Systematic Reviews and Meta-Analyses) flowchart showing the selection of sources of evidence. We were unable to retrieve 8 reports.

The process for the selection of sources of evidence is summarized in Figure 1 using the PRISMA flowchart (57). The literature search yielded 12,116 references, which was reduced to 7154 references after removal of duplicates. After the records had been screened by title and abstract, 326 reports were sought for retrieval. Three hundred eighteen reports were retrieved in full text and assessed for eligibility, and 99 reports were included in this scoping review (Table S3). The excluded papers that were based on full-text screening are listed in Table S1, along with reasons for exclusion.

### Number of Reports and Sample Sizes

**Figure 2:**
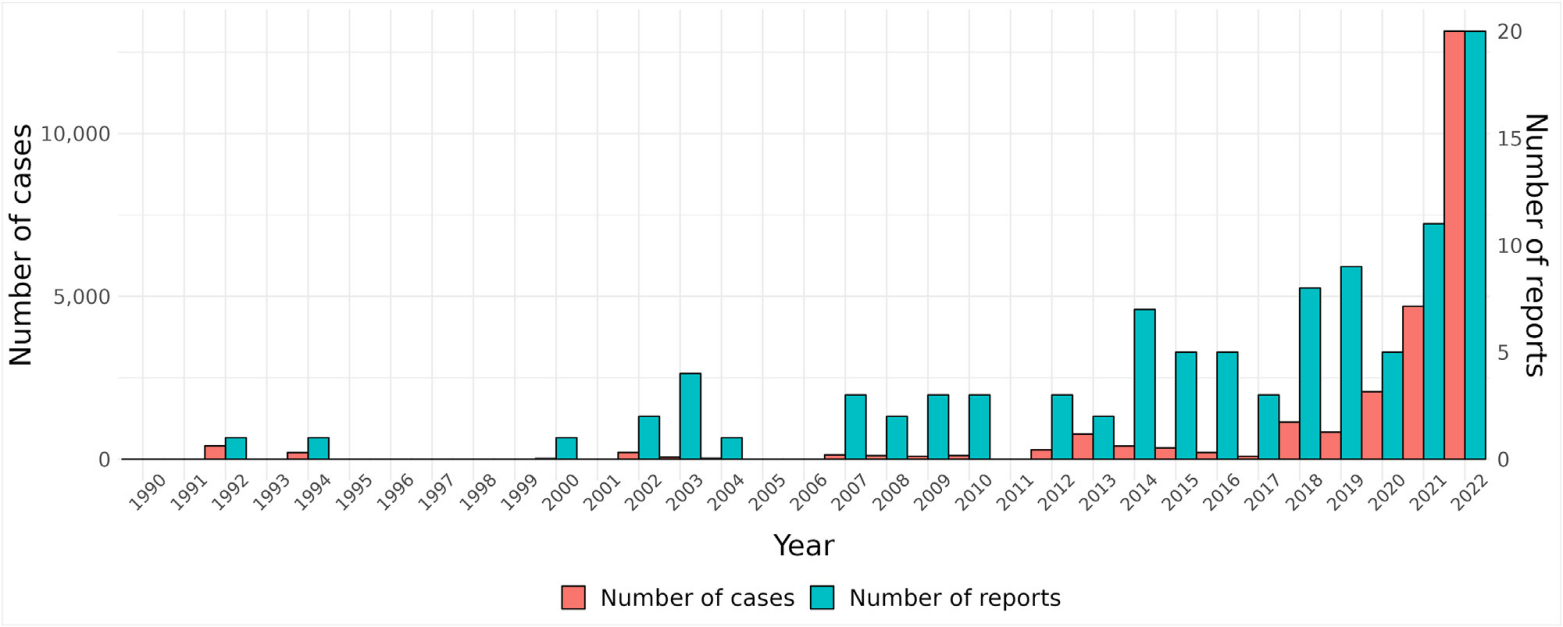
Number of reports on lipid biomarkers and bipolar disorder published from 1990 to 2022 and annual accumulated case sample sizes.

Both the number of reports published and sample sizes in these studies have increased steadily since 1990, with a particularly high rate during the last years (Figure 2). There were 2 reports published in the 1990s, while the annual number of reports increased from 3 in 2010 to 20 in 2022. In 1992, the total number of cases was 407, and in 2018 and 2019, the accumulated sample sizes were 1140 and 828, respectively. The accumulated sample sizes were considerably larger in the following years: 2073 cases in 2020, 4694 cases in 2021, and 13,145 cases in 2022.

### Laboratory Methods Used

**Figure 3:**
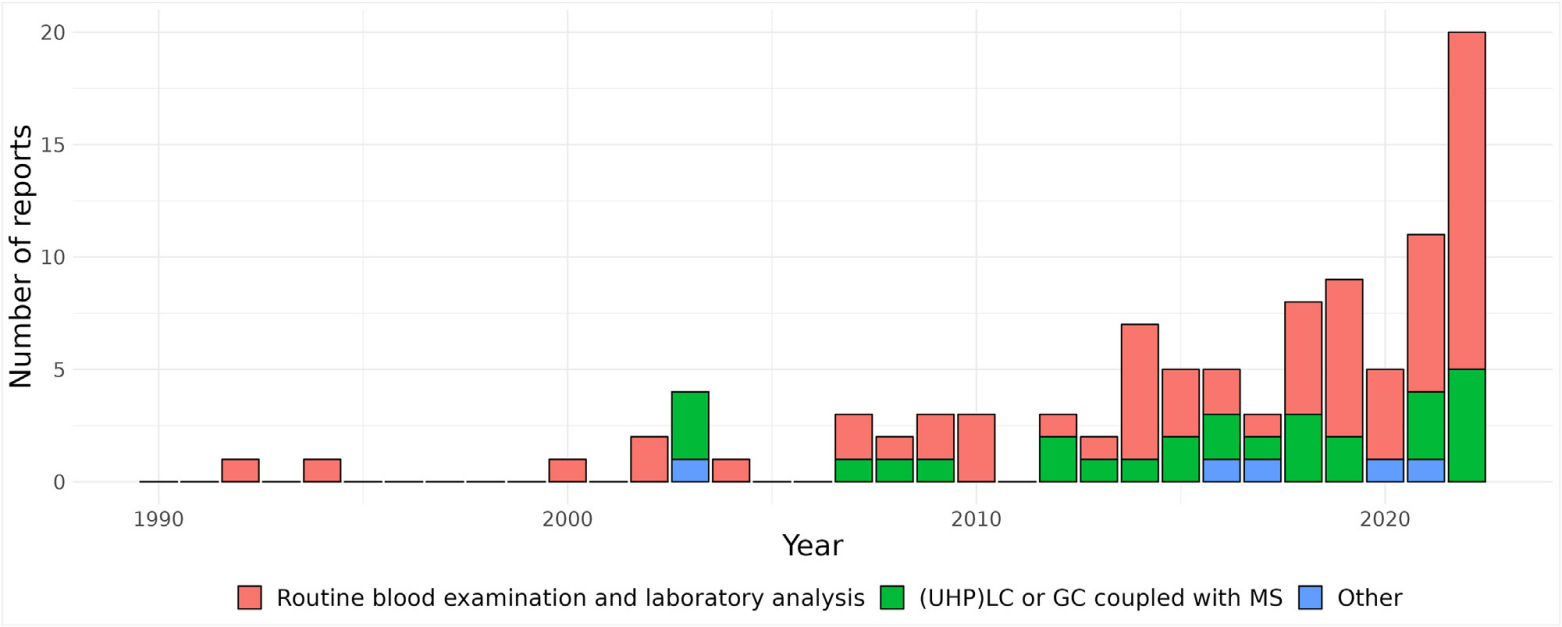
Frequency of different lipid measurement methods from 1990 to 2022. (UHP)LC, ultrahigh performance liquid chromatography; GC, gas chromatography; MS, mass spectrometry.

Figure 3 shows the distribution of methodologies used throughout the years. Routine laboratory assay has been the most used method for measuring lipid levels, with 15 reports using this method in 2022. Liquid chromatography and gas chromatography coupled with mass spectrometry, used for lipidomics, was first utilized in 3 reports in 2003 and in 5 reports in 2022.

### Lipids and Biological Samples Investigated

**Figure 4:**
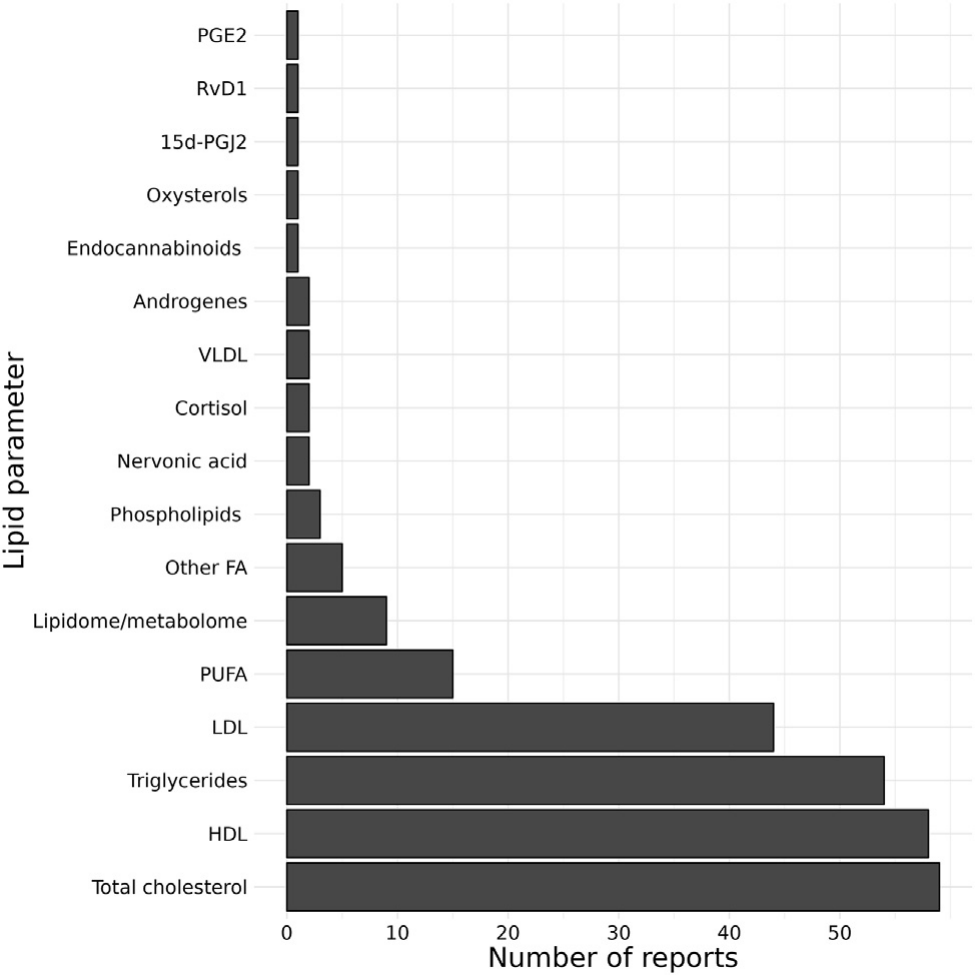
Lipid species and lipid classes researched from 1990 to 2022 and the number of reports investigating the respective lipid parameters. 15d-PGJ2, 15-deoxy-delta-12,14-prostaglandin J2; FA, fatty acid; HDL, high-density lipoprotein; LDL, low-density lipoprotein; PGE2, prostaglandin E2; PUFA, polyunsaturated fatty acid; RvD1, resolvin D1; VLDL, very low-density lipoprotein.

Lipid parameters investigated as potential biomarkers of BD since 1990 are shown in Figure 4. The most researched lipid measure was total cholesterol (TC), which was investigated in 59 reports. High-density lipoprotein (HDL) was the second most researched lipid measure (58 reports), while triglycerides (TG) and low-density lipoprotein (LDL) were reported in 54 and 44 papers, respectively. Lipids investigated in lipidomic studies are described in Supplement Note S3.

Only 2 studies did not investigate blood (whole blood, serum, plasma, or erythrocyte membranes) as the biological sample (Table S3). One of these papers reported on lipid biomarkers in cerebrospinal fluid (60) and the other reported on lipids in urine (61).

### Phenotypes Investigated

**Figure 5:**
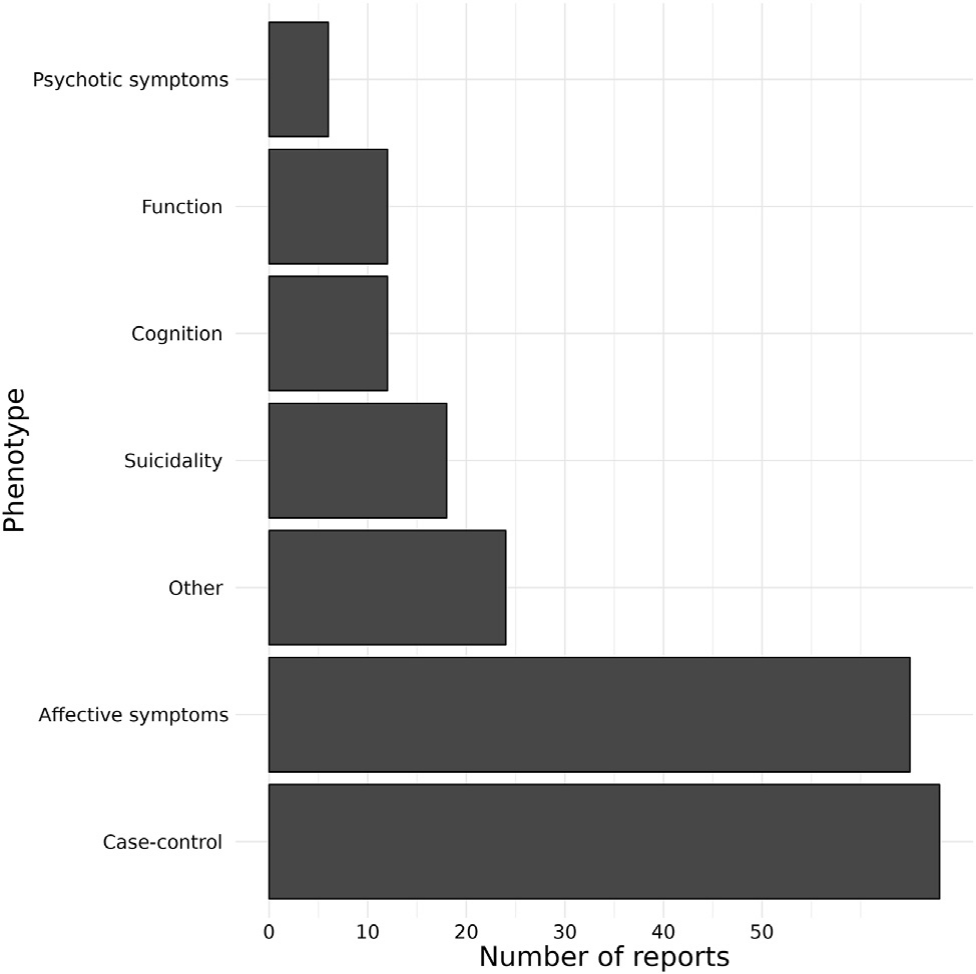
Phenotypes of bipolar disorder and the numbers of reports reporting on them. “Other” includes phenotypes like quality of life, personality factors, brain structure alterations, and more.

A variety of different phenotypes of BD have been investigated in relation to lipid biomarkers (Figure 5). The most researched subject was differences between BD cases and controls in 69 case-control studies. Notably, the control groups of several case-control studies consisted of patients with psychiatric disorders other than BD, such as schizophrenia or major depressive disorder. Affective symptoms were investigated in 65 publications, and other specific phenotypes researched include suicidality (thoughts, attempts, and completed suicides) in 18 papers, cognition in 12 papers, and function (measured using standard rating scales) and psychotic symptoms in 12 and 6 papers, respectively. Phenotypes such as brain structure alterations, personality factors, violence, and quality of life were also investigated in a total of 24 papers.

### Limitations of Studies

Small sample size, potential confounding effects, e.g., diet, physical activity, and medication use, and cross-sectional study design were frequently reported as study limitations. The limitations are fully summarized in Table S4 and Supplement Note S4.

### Key Study Findings

The results of lipid biomarker research were heterogeneous overall and to some extent contradictory. The majority of studies investigated standard lipids (TC, TG, LDL, HDL) and lipidomics. Several studies investigated more than one phenotype, and for reasons of clinical relevance, we present the findings based on separate phenotypes. A full summary and detailed description of study results for standard lipids are presented in Supplement Note S5. In the following section, we present trends in main results of standard lipids and summarize key findings of targeted and global lipidomics. The majority of studies using targeted lipidomics investigated fatty acids. Accordingly, the following summary of key findings from these studies is focused on fatty acids only. The complete data chart of key study findings can be found in Table S3.

#### Case-Control Comparisons

In the reviewed literature, 21 of the 38 reports that investigated TC, TG, LDL, or HDL in case-control studies found that patients with BD had at least one significantly altered lipid level (13,62, 63, 64, 65, 66, 67, 68, 69, 70, 71, 72, 73, 74, 75, 76, 77, 78, 79, 80, 81) compared with control participants. Of 14 reports that conducted targeted lipidomics/metabolomics analysis of erythrocyte membrane fatty acids or serum free fatty acids and included case-control status, 8 reports found significantly altered levels of fatty acids in cases compared with controls. Seven of them found lower levels of eicosapentaenoic acid (EPA) in patients with BD (82, 83, 84, 85, 86, 87, 88), while 1 publication reported higher levels of EPA in patients with BD than in control participants (89). Four reports showed lower docosahexaenoic acid (DHA) in patients with BD compared with control participants (84, 85, 86,89). Koga *et al.* (84) reported significantly increased levels of all n-6 polyunsaturated fatty acids (PUFAs) in patients with BD compared with control participants. In case-control studies that involved subjects with BD and patients with other psychiatric disorders, 3 of 19 studies found decreased levels of TC, LDL, nervonic acid, and DHA in BD compared with schizophrenia or major depressive disorder (90, 91, 92), while 1 study found higher TC levels in BD than in unipolar depression (67).

#### Depressive Episode

Ten of 31 reports that assessed levels of TC, TG, LDL, and HDL in patients with BD experiencing a depressive episode found that at least one of the lipids was altered in cases (22,64,67,78,80,90,93, 94, 95, 96). Notably, 8 studies showed that depressive episodes were associated with increased levels of at least one lipid (22,67,78,80,90,94, 95, 96). In targeted omics of erythrocyte membrane fatty acids and serum free fatty acids, 3 of the 7 studies that investigated depressive episodes in BD found significantly lower levels of DHA in patients with BD (85,86,97), while in the 4 other reports, there were either no associations with depressive symptoms or associations that were not replicated in other studies.

#### Manic Episode

Of 29 reports that investigated levels of TC, TG, LDL, and HDL in patients with mania, 10 found at least one significantly altered lipid in patients with BD (13,20,22,62,64,77,78,80,98,99). Moreover, all 10 studies found decreases of at least one lipid. Six reports investigated erythrocyte membrane fatty acids or serum free fatty acids using targeted omics methods, and 4 of these found significantly altered lipid levels (85,86,89,97,100,101). Three of these 4 reports found decreased DHA to be associated with manic episodes in patients with BD (85,86,100).

#### Suicidality

Six of the 18 reports that investigated TC, TG, LDL, or HDL in bipolar patients with suicidal behavior found that at least one of these lipids was significantly altered. Two of the reports found significantly increased lipid levels in patients who showed suicidality (79,102), while 4 found decreased lipids to be associated with suicidal tendencies (103, 104, 105, 106). Furthermore, Saunders *et al.* (88) found negative correlations between serum EPA and suicidality.

#### Global Omics

Due to the nature of global lipidomics and metabolomics, the results of reports utilizing these techniques are heterogeneous. However, there are some classes of lipids that tend to display potential as biomarkers for BD throughout the literature (Table 1). Four of the 9 reports that used omics methods found glycerophospholipids to be altered in BD (44,107, 108, 109), and 4 publications also reported altered levels of sphingolipids in BD (44,108, 109, 110). Four studies reported altered glycerolipids (44,108, 109, 110), and 3 reported altered levels of free fatty acids (91,108,111). Avigdor *et al.* (112) and Du *et al.* (107) also found altered levels of platelet-activating factor to be associated with BD.

**Table 1:** Reports Using Global Lipidomics and Metabolomics to Identify Lipids as Potential Biomarkers

Publication	Potential Lipid Biomarkers for BD	Method	Phenotype	Body Fluid
Avigdor *et al.*, 2021 (112)	PAF (phospholipid)	UHPLC-MS	CC, FEP	Plasma
Brunkhorst-Kanaan *et al.*, 2019 (110)	Glycerolipids, ceramides (sphingolipids)	LC-MS	CC, DE, MaE, MiE	Plasma
Du *et al.*, 2022 (107)	Chenodeoxycholic acid (steroid), PAF, glycerophospholipids	UHPLC-MS	CC	Serum
Guo *et al.*, 2022 (108)	Fatty acids, sphingolipids, saccharolipids, prenol lipids, glycerolipids, glycerophospholipids	UHPLC-MS	CC, DE, MaE	Plasma
Kageyama *et al.*, 2018 (91)	None	LC-MS and GC-MS	CC, DE, MaE, remitted	Plasma
Ren *et al.*, 2021 (61)	Glycerol	H-NMR	CC, DE	Urine
Ribeiro *et al.*, 2017 (44)	Glycerophospholipids, glycerolipids, sphingolipids	UHPLC-MS	CC, Euthymia	Serum
Ribeiro *et al.*, 2022 (111)	Cholesterol, fatty acids	GC-MS	CC	Serum
Zhang *et al.*, 2022 (109)	Glycerolipids, glycerophospholipids, sphingolipids	LC-MS	CC	Plasma

BP, bipolar disorder; CC, case-control; DE, depressive episode; FEP, first-episode psychosis; GC, gas chromatography; H-NMR, nuclear magnetic resonance; LC, liquid chromatography; MaE, manic episode; MiE, mixed episode; MS, mass spectrometry; PAF, platelet-activating factor; UHPLC, ultrahigh performance liquid chromatography.

## Discussion

In the current scoping review, we sought to summarize and describe the research on lipid biomarkers in BD since 1990 and identify important future directions for this research. Through rigorous screening and study selection, 99 papers were included in this review. Notably, a number of specifying alterations were applied to the eligibility criteria for the full-text screening, as the reviewers found it appropriate (Supplement Note S1).

We identified a large increase in both annual study numbers and annual accumulated sample sizes throughout the years since 1990. The mean sample size per report published has also increased considerably, and the research field has clearly gained traction, especially during the last decade (13, 14, 15,113,114).

The laboratory methods used to measure lipid levels of subjects with BD have been stable, with frequent use of standard clinical laboratory measurement.

The 4 commonly studied lipid parameters (TC, HDL, TG, and LDL) are routine markers in clinical practice and therefore are easily available. Demonstrating associations between BD and these lipid levels may therefore be of value when considering cost-effectiveness. There are several theories connecting these 4 lipids to the pathogenesis of BD, such as the cholesterol-serotonin hypothesis (115, 116, 117, 118) and the antioxidative effects of HDL (18,119,120). We found that 68 studies had assessed these 4 lipids as potential biomarkers for BD. Overall, few studies showed replicable associations between the levels of these lipids and BD phenotypes, leaving the field inconclusive. Notably, a tendency of direction of effect was found within studies finding associations; studies of depressive episodes tended to be associated with increased levels of lipids, while manic episodes tended to be associated with decreased lipids. This may be explained by the different energy expenditures that take place in depression and mania (121,122).

However, the study of TC, TG, LDL, and HDL as biomarkers is challenging due to many possible confounders such as food intake, comorbid somatic disorders, and medication use. In fact, psychotropic drugs used to treat BD are known to affect lipid metabolism and may lead to altered lipid parameters (17,123,124). In this scoping review, we aimed to assess the most pathophysiologically relevant changes in lipid levels in BD. Therefore, we excluded studies that investigated lipid alterations specifically related to medication. However, many of the studies included subjects that were taking medication that potentially confounded the results. The lack of rigorous study methods to account for the use of medication was frequently reported as a limitation.

Lipidomics and metabolomics are relatively new and promising approaches for discovering lipid biomarkers (125,126). The use of targeted and global lipidomics in the research for lipid biomarkers in BD is slightly increasing, with 5 published papers using this method in 2022 (44,107, 108, 109,127). In targeted lipidomics, a predetermined set of lipids is chosen for analysis, and in our review, we found that studies investigated a variety of different lipid classes. Therefore, comparison between these studies is challenging due to the heterogeneity of analyzed lipids. Moreover, most studies that utilized targeted lipidomics found an association between lipids investigated and BD phenotypes. Fatty acids were assessed in the majority of studies, and the most consistent findings were related to levels of EPA and DHA. The large pool of research suggesting a key inflammatory component in BD may explain why PUFAs have been frequently investigated (119,128, 129, 130). n-6 PUFAs in particular have been shown to be proinflammatory (131), which can explain the elevated levels of all n-6 PUFAs in cases of BD compared with controls found by Koga *et al.* (84). With the use of global lipidomics, glycerophospholipids, sphingolipids, glycerolipids, and fatty acids were found as potential biomarkers across studies. If replicated, these findings could be explained by their biological functions in the brain, metabolism, and immune system (132, 133, 134).

Lipidomics is an effective way of investigating potential biomarkers because it requires miniscule amounts of biological sample and can detect and quantify thousands of molecules in a single analysis run (135,136). This method enables hypothesis generation, which can help direct more targeted investigations later. Lipidomics are used to investigate lipids and their metabolites, which not only allows for identification of specific lipid molecules that are altered in BD, but also provides information on a systems level, reflecting the exact steps in the metabolic pathways that are dysregulated (125,126). However, these analyses are costly and time consuming, making this measurement technique difficult to attain for many research facilities (137,138). Furthermore, the reliability and validity of lipidomic analyses may be somewhat inferior to standard laboratory methods because they are more vulnerable to measurement error (139). Nevertheless, future research may benefit from pivoting toward the use of newer techniques. In the case of lipidomics technology, predefined lipids should be explored using targeted lipidomics in large cohorts, based on results from discovery studies with hypothesis-generating global lipidomics, thereby conducting cost-effective and replicable studies. The reliability of the analyses could also be maximized by adhering to preregistered protocols for lipidomic analysis (140). This could also be combined with causal inference methods like Mendelian randomization to guide future clinical trials of lipid-altering medication (141).

Only 2 of the papers included in this review analyzed body fluids other than blood—one investigated urine lipid levels, and the other investigated cerebrospinal fluid levels. Although readily available for sampling, urine contains very small amounts of lipids in the absence of kidney disease, which may explain the lack of studies investigating urine lipids as biomarkers for BD (142). In the case of cerebrospinal fluid, collection of this biological fluid requires costly procedures that pose risks for patients and are not easily accessible in most health institutions (143).

The high rate of comorbidity and heterogeneous symptom profiles has led to investigations of the development of new and improved diagnostic criteria and classification of symptoms based on pathophysiological state markers with better prognostic properties (144,145). Therefore, finding biomarkers for affective symptoms and other bipolar phenomenology may be more beneficial than case-control studies because there is considerable overlap between the diagnostic criteria for severe mental disorders, such as major depressive disorder and other psychotic disorders. For comparison, altered levels of PUFAs (38,45,47,48), phospholipids (45,48), carnitines, and free fatty acids (45,46) have been suggested as potential markers of schizophrenia, while altered levels of PUFAs (49, 50, 51), HDL (50,146), TG, and phospholipids (50,51) have been suggested to distinguish patients with major depressive disorder from control participants. Notably, lower levels of TC have been found to be associated with suicidal behavior in both schizophrenia and major depressive disorder (147,148). Furthermore, research on lipid biomarkers associated with variation in affective symptoms is important because it is crucial to distinguish between state or trait markers of the disease (149). The obvious commonly altered eating patterns and levels of physical activity in depressive versus manic states increase the need for complex study designs that involve investigating lipid levels over time. However, few of the case-control studies and studies on affective symptoms found in our review were longitudinal, making evaluation of causal relationships between altered lipid levels and psychiatric phenomenology challenging. In future efforts, a longitudinal design should be used to investigate the prognostic value of lipid biomarkers.

### Limitations of Review

Critical appraisal of methods and full synthesis of results are beyond the scope of this review design, with the associated possibility that we included studies of poor method quality. Furthermore, the descriptive presentation of results of the large number of reports included could leave key points biased. Moreover, genetic studies that address causal inference with methods such as Mendelian randomization were not included. To address the research question of to what extent lipid biomarkers are currently found in BD, a systematic review could be beneficial. However, the divergent study methods and research gaps found in this review suggest that future efforts should focus on alignment of study methods and minimizing frequent limitations. An important strength is the comprehensive literature search in 4 separate bibliographic databases and inclusion of a large body of literature.

### Conclusions

The current scoping review of the literature on lipid biomarkers in BD shows a large increase in the amount of research that has been conducted during the last 3 decades, with developments in technology and growing knowledge in medical biochemistry. Moreover, the understanding of the role of lipids in pathophysiological mechanisms has even broader implications if the use of lipids as medication is thought to have potential psychopharmacological properties. Taken together, the results of the research on lipid biomarkers in BD vary greatly in terms of phenotypes investigated and study designs, leading to divergent findings. The reported limitations such as modest sample sizes; potential confounders like physical activity, nutritional status, and socioeconomic status; subjects taking medication; and nonlongitudinal study designs should be addressed in future research. The development of lipids as reliable prognostic and diagnostic biomarkers relies on consistent study methods across cohorts, with robust sample sizes, and the utilization of newer laboratory techniques such as lipidomics.

## Supplementary Material

Supplemental Data

Key Resources Table

## References

[1] Grande I., Berk M., Birmaher B., Vieta E. (2016). Bipolar disorder. Lancet.

[2] Cardoso G., Xavier M., Vilagut G., Petukhova M., Alonso J., Kessler R.C., Caldas-de-Almeida J.M. (2017). Days out of role due to common physical and mental conditions in Portugal: Results from the WHO World Mental Health Survey. BJPsych Open.

[3] Crump C., Sundquist K., Winkleby M.A., Sundquist J. (2013). Comorbidities and mortality in bipolar disorder: A Swedish national cohort study. JAMA Psychiatry.

[4] Lozano R., Naghavi M., Foreman K., Lim S., Shibuya K., Aboyans V. (2012). Global and regional mortality from 235 causes of death for 20 age groups in 1990 and 2010: A systematic analysis for the Global Burden of Disease Study 2010. Lancet.

[5] Merikangas K.R., Jin R., He J.P., Kessler R.C., Lee S., Sampson N.A. (2011). Prevalence and correlates of bipolar spectrum disorder in the world mental health survey initiative. Arch Gen Psychiatry.

[6] Carvalho A.F., Firth J., Vieta E. (2020). Bipolar disorder. N Engl J Med.

[7] Paus T., Keshavan M., Giedd J.N. (2008). Why do many psychiatric disorders emerge during adolescence?. Nat Rev Neurosci.

[8] Päären A., Bohman H., Von Knorring L., Olsson G., Von Knorring A.L., Jonsson U. (2014). Early risk factors for adult bipolar disorder in adolescents with mood disorders: A 15-year follow-up of a community sample. BMC Psychiatry.

[9] Phillips M.L., Kupfer D.J. (2013). Bipolar disorder diagnosis: Challenges and future directions. Lancet.

[10] Etain B., Mathieu F., Rietschel M., Maier W., Albus M., Mckeon P. (2006). Genome-wide scan for genes involved in bipolar affective disorder in 70 European families ascertained through a bipolar type I early-onset proband: Supportive evidence for linkage at 3p14. Mol Psychiatry.

[11] Gurling H., Smyth C., Kalsi G., Moloney E., Rifkin L., O’Neill J. (1995). Linkage findings in bipolar disorder. Nat Genet.

[12] Axelson D., Goldstein B., Goldstein T., Monk K., Yu H., Hickey M.B. (2015). Diagnostic precursors to bipolar disorder in offspring of parents with bipolar disorder: A longitudinal study. Am J Psychiatry.

[13] Pae C.U., Kim J.J., Lee S.J., Lee C., Paik I.H., Lee C.U. (2004). Aberration of cholesterol level in first-onset bipolar I patients. J Affect Disord.

[14] Mcnamara R.K., Jandacek R., Rider T., Tso P., Stanford K.E., Hahn C.G., Richtand N.M. (2008). Deficits in docosahexaenoic acid and associated elevations in the metabolism of arachidonic acid and saturated fatty acids in the postmortem orbitofrontal cortex of patients with bipolar disorder. Psychiatry Res.

[15] Wysokiński A., Strzelecki D., Kłoszewska I. (2015). Levels of triglycerides, cholesterol, LDL, HDL and glucose in patients with schizophrenia, unipolar depression and bipolar disorder. Diabetes Metab Syndr.

[16] ÜÇOK A., GAEBEL W. (2008). Side effects of atypical antipsychotics: A brief overview. World Psychiatry.

[17] Reynolds G.P., Kirk S.L. (2010). Metabolic side effects of antipsychotic drug treatment – Pharmacological mechanisms. Pharmacol Ther.

[18] Steckert A.V., Valvassori S.S., Moretti M., Dal-Pizzol F., Quevedo J. (2010). Role of oxidative stress in the pathophysiology of bipolar disorder. Neurochem Res.

[19] Kuloglu M., Ustundag B., Atmaca M., Canatan H., Tezcan A.E., Cinkilinc N. (2002). Lipid peroxidation and antioxidant enzyme levels in patients with schizophrenia and bipolar disorder. Cell Biochem Funct.

[20] Huang Y.J., Tsai S.Y., Chung K.H., Chen P.H., Huang S.H., Kuo C.J. (2018). State-dependent alterations of lipid profiles in patients with bipolar disorder. Int J Psychiatry Med.

[21] Pan P., Qiu Y., Teng Z., Li S., Huang J., Xiang H. (2021). Increased global-brain functional connectivity is associated with dyslipidemia and cognitive impairment in first-episode, drug-naive patients with bipolar disorder. Neural Plast.

[22] Fusar-Poli L., Amerio A., Cimpoesu P., Natale A., Salvi V., Zappa G. (2020). Lipid and glycemic profiles in patients with bipolar disorder: Cholesterol levels are reduced in mania. Medicina (Kaunas).

[23] Enger C., Jones M.E., Kryzhanovskaya L., Doherty M., McAfee A.T. (2013). Risk of developing diabetes and dyslipidemia among adolescents with bipolar disorder or schizophrenia. Int J Adolesc Med Health.

[24] Hennekens C.H., Hennekens A.R., Hollar D., Casey D.E. (2005). Schizophrenia and increased risks of cardiovascular disease. Am Heart J.

[25] Munkholm K., Peijs L., Kessing L.V., Vinberg M. (2014). Reduced mRNA expression of PTGDS in peripheral blood mononuclear cells of rapid-cycling bipolar disorder patients compared with healthy control subjects. Int J Neuropsychopharmacol.

[26] Skosnik P.D., Yao J.K. (2003). From membrane phospholipid defects to altered neurotransmission: Is arachidonic acid a nexus in the pathophysiology of schizophrenia?. Prostaglandins Leukot Essent Fatty Acids.

[27] Ikeda M., Takahashi A., Kamatani Y., Okahisa Y., Kunugi H., Mori N. (2018). A genome-wide association study identifies two novel susceptibility loci and trans population polygenicity associated with bipolar disorder. Mol Psychiatry.

[28] Rødevand L., Bahrami S., Frei O., Chu Y., Shadrin A., O’Connell K.S. (2021). Extensive bidirectional genetic overlap between bipolar disorder and cardiovascular disease phenotypes. Transl Psychiatry.

[29] Steen V.M., Skrede S., Polushina T., López M., Andreassen O.A., Fernø J., Hellard S.L. (2017). Genetic evidence for a role of the SREBP transcription system and lipid biosynthesis in schizophrenia and antipsychotic treatment. Eur Neuropsychopharmacol.

[30] Salagre E., Fernandes B.S., Dodd S., Brownstein D.J., Berk M. (2016). Statins for the treatment of depression: A meta-analysis of randomized, double-blind, placebo-controlled trials. J Affect Disord.

[31] Yatham M.S., Yatham K.S., Ravindran A.V., Sullivan F. (2019). Do statins have an effect on depressive symptoms? A systematic review and meta-analysis. J Affect Disord.

[32] Redlich C., Berk M., Williams L.J., Sundquist J., Sundquist K., Li X. (2014). Statin use and risk of depression: A Swedish national cohort study. BMC Psychiatry.

[33] Jiang J.C., Hu C., McIntosh A.M., Shah S. (2023). Investigating the potential anti-depressive mechanisms of statins: A transcriptomic and Mendelian randomization analysis. Transl Psychiatry.

[34] Fernandes B.S., Williams L.M., Steiner J., Leboyer M., Carvalho A.F., Berk M. (2017). The new field of ‘precision psychiatry.’. BMC Med.

[35] Rodrigues-Amorim D., Rivera-Baltanás T., López M., Spuch C., Olivares J.M., Agís-Balboa R.C. (2017). Schizophrenia: A review of potential biomarkers. J Psychiatr Res.

[36] Pickard B.S. (2015). Schizophrenia biomarkers: Translating the descriptive into the diagnostic. J Psychopharmacol.

[37] Carvalho A.F., Köhler C.A., Fernandes B.S., Quevedo J., Miskowiak K.W., Brunoni A.R. (2016). Bias in emerging biomarkers for bipolar disorder. Psychol Med.

[38] Davison J., O’Gorman A., Brennan L., Cotter D.R. (2018). A systematic review of metabolite biomarkers of schizophrenia. Schizophr Res.

[39] Teixeira A.L., Colpo G.D., Fries G.R., Bauer I.E., Selvaraj S. (2019). Biomarkers for bipolar disorder: Current status and challenges ahead. Expert Rev Neurother.

[40] Frey B.N., Andreazza A.C., Houenou J., Jamain S., Goldstein B.I., Frye M.A. (2013). Biomarkers in bipolar disorder: A positional paper from the International Society for Bipolar Disorders Biomarkers Task Force. Aust N Z J Psychiatry.

[41] Sigitova E., Fišar Z., Hroudová J., Cikánková T., Raboch J. (2017). Biological hypotheses and biomarkers of bipolar disorder. Psychiatry Clin Neurosci.

[42] Muneer A. (2016). Bipolar disorder: Role of inflammation and the development of disease biomarkers. Psychiatry Investig.

[43] Schwarz E., Prabakaran S., Whitfield P., Major H., Leweke F.M., Koethe D. (2008). High throughput lipidomic profiling of schizophrenia and bipolar disorder brain tissue reveals alterations of free fatty acids, phosphatidylcholines, and ceramides. J Proteome Res.

[44] Ribeiro H.C., Klassen A., Pedrini M., Carvalho M.S., Rizzo L.B., Noto M.N. (2017). A preliminary study of bipolar disorder type I by mass spectrometry-based serum lipidomics. Psychiatry Res.

[45] Zhuo C., Hou W., Tian H., Wang L., Li R. (2020). Lipidomics of the brain, retina, and biofluids: From the biological landscape to potential clinical application in schizophrenia. Transl Psychiatry.

[46] Liu Y., Song X., Liu X., Pu J., Gui S., Xu S. (2021). Alteration of lipids and amino acids in plasma distinguish schizophrenia patients from controls: A targeted metabolomics study. Psychiatry Clin Neurosci.

[47] Fenton W.S., Hibbeln J., Knable M. (2000). Essential fatty acids, lipid membrane abnormalities, and the diagnosis and treatment of schizophrenia. Biol Psychiatry.

[48] Berger G.E., Wood S.J., Pantelis C., Velakoulis D., Wellard R.M., Mcgorry P.D. (2002). Implications of lipid biology for the pathogenesis of schizophrenia. Aust N Z J Psychiatry.

[49] Müller C.P., Reichel M., Mühle C., Rhein C., Gulbins E., Kornhuber J. (2015). Brain membrane lipids in major depression and anxiety disorders. Biochim Biophys Acta.

[50] Parekh A., Smeeth D., Milner Y., Thure S. (2017). The role of lipid biomarkers in major depression. Healthcare (Basel).

[51] Walther A., Cannistraci C.V., Simons K., Durán C., Gerl M.J., Wehrli S., Kirschbaum C. (2018). Lipidomics in major depressive disorder. Front Psychiatry.

[52] Tricco A.C., Lillie E., Zarin W., O’Brien K.K., Colquhoun H., Levac D. (2018). PRISMA extension for scoping reviews (PRISMA-ScR): Checklist and explanation. Ann Intern Med.

[53] Arksey H., O’Malley L. (2005). Scoping studies: Towards a methodological framework. Int J Soc Res Methodol.

[54] Peters M.D.J., Marnie C., Tricco A.C., Pollock D., Munn Z., Alexander L. (2020). Updated methodological guidance for the conduct of scoping reviews. JBI Evid Synth.

[55] Hiller J.K. (2021). Lipid biomarkers in bipolar disorder: Protocol for a scoping review. https://osf.io/4k5nd.

[56] Moher D., Shamseer L., Clarke M., Ghersi D., Liberati A., Petticrew M. (2015). Preferred reporting items for systematic review and meta-analysis protocols (PRISMA-P) 2015 statement. Syst Rev.

[57] Page M.J., Mckenzie J.E., Bossuyt P.M., Boutron I., Hoffmann T.C., Mulrow C.D. (2021). The PRISMA 2020 statement: An updated guideline for reporting systematic reviews. Syst Rev.

[58] Ouzzani M., Hammady H., Fedorowicz Z., Elmagarmid A. (2016). Rayyan-a web and mobile app for systematic reviews. Syst Rev.

[59] Wickham H. (2016).

[60] Kageyama Y., Deguchi Y., Hattori K., Yoshida S., Goto Y.I., Inoue K., Kato T. (2021). Nervonic acid level in cerebrospinal fluid is a candidate biomarker for depressive and manic symptoms: A pilot study. Brain Behav.

[61] Ren Y., Chen Z.Z., Sun X.L., Duan H.J., Tian J.S., Wang J.Y., Yang H. (2021). Metabolomic analysis to detect urinary molecular changes associated with bipolar depression. Neurosci Lett.

[62] Atmaca M., Kuloglu M., Tezcan E., Ustundag B., Bayik Y. (2002). Serum leptin and cholesterol levels in patients with bipolar disorder. Neuropsychobiology.

[63] Dalkner N., Bengesser S.A., Birner A., Fellendorf F.T., Fleischmann E., Großschädl K. (2021). Metabolic syndrome impairs executive function in bipolar disorder. Front Neurosci.

[64] De Berardis D., Conti C.M., Campanella D., Carano A., Scali M., Valchera A. (2008). Evaluation of C-reactive protein and total serum cholesterol in adult patients with bipolar disorder. Int J Immunopathol Pharmacol.

[65] Ezzaher A., Mouhamed D.H., Mechri A., Araoud M., Neffati F., Douki W. (2010). Lower paraoxonase 1 activity in Tunisian bipolar I patients. Ann Gen Psychiatry.

[66] Fürtjes A.E., Coleman J.R.I., Tyrrell J., Lewis C.M., Hagenaars S.P. (2021). Associations and limited shared genetic aetiology between bipolar disorder and cardiometabolic traits in the UK Biobank. Psychol Med.

[67] Ghaemi S.N., Shields G.S., Hegarty J.D., Goodwin F.K. (2000). Cholesterol levels in mood disorders: High or low?. Bipolar Disord.

[68] Glueck C.J., Tieger M., Kunkel R., Hamer T., Tracy T., Speirs J. (1994). Hypocholesterolemia and affective disorders. Am J Med Sci.

[69] Guidara W., Messedi M., Maalej M., Naifar M., Khrouf W., Grayaa S. (2021). Plasma oxysterols: Altered level of plasma 24-hydroxycholesterol in patients with bipolar disorder. J Steroid Biochem Mol Biol.

[70] Kasak M., Ceylan M.F., Hesapcioglu S.T., Senat A., Erel Ö. (2022). Peroxisome proliferator-activated receptor gamma (PPARγ) levels in adolescent with bipolar disorder and their relationship with metabolic parameters. J Mol Neurosci.

[71] Kennedy K.G., Islam A.H., Grigorian A., Fiksenbaum L., Mitchell R.H.B., McCrindle B.W. (2021). Elevated lipids are associated with reduced regional brain structure in youth with bipolar disorder. Acta Psychiatr Scand.

[72] Naiberg M.R., Newton D.F., Collins J.E., Bowie C.R., Goldstein B.I. (2016). Impulsivity is associated with blood pressure and waist circumference among adolescents with bipolar disorder. J Psychiatr Res.

[73] Naiberg M.R., Newton D.F., Collins J.E., Dickstein D.P., Bowie C.R., Goldstein B.I. (2016). Elevated triglycerides are associated with decreased executive function among adolescents with bipolar disorder. Acta Psychiatr Scand.

[74] Nunes S.O., Piccoli de Melo L.G., Pizzo de Castro M.R., Barbosa D.S., Vargas H.O., Berk M., Maes M. (2015). Atherogenic index of plasma and atherogenic coefficient are increased in major depression and bipolar disorder, especially when comorbid with tobacco use disorder. J Affect Disord.

[75] Porcu M., Urbano M.R., Verri W.A., Machado R.C.R., Vargas H.O., Nunes S.O.V. (2022). Comparison of the severity of depressive and anxiety symptoms, biomarkers, and childhood trauma among bipolar smokers and non-smokers, and controls. J Affect Disord Rep.

[76] Qiu Y., Li S., Teng Z., Tan Y., Xu X., Yang M. (2022). Association between abnormal glycolipid level and cognitive dysfunction in drug-naive patients with bipolar disorder. J Affect Disord.

[77] Sagud M., Mihaljevic-Peles A., Pivac N., Jakovljevic M., Muck-Seler D. (2007). Platelet serotonin and serum lipids in psychotic mania. J Affect Disord.

[78] Sagud M., Mihaljevic-Peles A., Pivac N., Jakovljevic M., Muck-Seler D. (2009). Lipid levels in female patients with affective disorders. Psychiatry Res.

[79] Su M., Li E., Tang C., Zhao Y., Liu R., Gao K. (2019). Comparison of blood lipid profile/thyroid function markers between unipolar and bipolar depressed patients and in depressed patients with anhedonia or suicidal thoughts. Mol Med.

[80] Wei Y., Wang T., Li G., Feng J., Deng L., Xu H. (2022). Investigation of systemic immune-inflammation index, neutrophil/high-density lipoprotein ratio, lymphocyte/high-density lipoprotein ratio, and monocyte/high-density lipoprotein ratio as indicators of inflammation in patients with schizophrenia and bipolar disorder. Front Psychiatry.

[81] Wulsin L.R., Blom T.J., Durling M., Welge J.A., DelBello M.P., Adler C.M. (2018). Cardiometabolic risks and omega-3 index in recent-onset bipolar I disorder. Bipolar Disord.

[82] Evans S.J., Ringrose R.N., Harrington G.J., Mancuso P., Burant C.F., McInnis M.G. (2014). Dietary intake and plasma metabolomic analysis of polyunsaturated fatty acids in bipolar subjects reveal dysregulation of linoleic acid metabolism. J Psychiatr Res.

[83] Evans S.J., Assari S., Harrington G.J., Chang Y.W., Burant C.F., McInnis M.G. (2015). Plasma linoleic acid partially mediates the association of bipolar disorder on self-reported mental health scales. J Psychiatr Res.

[84] Koga N., Ogura J., Yoshida F., Hattori K., Hori H., Aizawa E. (2019). Altered polyunsaturated fatty acid levels in relation to proinflammatory cytokines, fatty acid desaturase genotype, and diet in bipolar disorder. Transl Psychiatry.

[85] McNamara R.K., Jandacek R., Tso P., Blom T.J., Welge J.A. (2016). Adolescents with or at ultra-high risk for bipolar disorder exhibit erythrocyte docosahexaenoic acid and eicosapentaenoic acid deficits: a candidate prodromal risk biomarker. Early Interv Psychiatry.

[86] McNamara R.K., Moser A.B., Jones R.I., Jandacek R., Patino L.R., Strawn J.R. (2016). Familial risk for bipolar disorder is not associated with impaired peroxisomal function: Dissociation from docosahexaenoic acid deficits. Psychiatry Res.

[87] Ranjekar P.K., Hinge A., Hegde M.V., Ghate M., Kale A., Sitasawad S. (2003). Decreased antioxidant enzymes and membrane essential polyunsaturated fatty acids in schizophrenic and bipolar mood disorder patients. Psychiatry Res.

[88] Saunders E.F.H., Ramsden C.E., Sherazy M.S., Gelenberg A.J., Davis J.M., Rapoport S.I. (2016). Omega-3 and Omega-6 polyunsaturated fatty acids in bipolar disorder: A review of biomarker and treatment studies. J Clin Psychiatry.

[89] Pomponi M., Janiri L., La Torre G., Di Stasio E., Di Nicola M., Mazza M. (2013). Plasma levels of n-3 fatty acids in bipolar patients: Deficit restricted to DHA. J Psychiatr Res.

[90] Gohar S.M., Dieset I., Steen N.E., Mørch R.H., Iversen T.S., Steen V.M. (2019). Association between serum lipid levels, osteoprotegerin and depressive symptomatology in psychotic disorders. Eur Arch Psychiatry Clin Neurosci.

[91] Kageyama Y., Kasahara T., Nakamura T., Hattori K., Deguchi Y., Tani M. (2018). Plasma nervonic acid is a potential biomarker for major depressive disorder: A pilot study. Int J Neuropsychopharmacol.

[92] Sublette M.E., Bosetti F., DeMar J.C., Ma K., Bell J.M., Fagin-Jones S. (2007). Plasma free polyunsaturated fatty acid levels are associated with symptom severity in acute mania. Bipolar Disord.

[93] Birdsall J.W., Schmitz S.L., Abosi O.J., DuBose L.E., Pierce G.L., Fiedorowicz J.G. (2019). Inflammatory and vascular correlates of mood change over 8 weeks. Heart Mind (Mumbai).

[94] Chung K.H., Tsai S.Y., Lee H.C. (2007). Mood symptoms and serum lipids in acute phase of bipolar disorder in Taiwan. Psychiatry Clin Neurosci.

[95] Congio A.C., Rossaneis A.C., Verri W.A., Urbano M.R., Nunes S.O.V. (2022). Childhood trauma, interleukin-17, C-reactive protein, metabolism, and psychosocial functioning in bipolar depression. J Affect Disord Rep.

[96] Richter N., Juckel G., Assion H.J. (2010). Metabolic syndrome: A follow-up study of acute depressive inpatients. Eur Arch Psychiatry Clin Neurosci.

[97] Clayton E.H., Hanstock T.L., Hirneth S.J., Kable C.J., Garg M.L., Hazell P.L. (2008). Long-chain omega-3 polyunsaturated fatty acids in the blood of children and adolescents with juvenile bipolar disorder. Lipids.

[98] Cassidy F., Carroll B.J. (2002). Hypocholesterolemia during mixed manic episodes. Eur Arch Psychiatry Clin Neurosci.

[99] Erzin G., Aydemir M.Ç., Yüksel R.N., Tatlıdil Yaylacı E., Çakır B., Sezer S., Göka E. (2019). Serum 15-d-PGJ2 and PPARgamma levels are reduced in manic episode of bipolar disorder while IL-4 levels are not affected. Psychiatry Clin Psychopharmacol.

[100] Chiu C.C., Huang S.Y., Su K.P., Lu M.L., Huang M.C., Chen C.C., Shen W.W. (2003). Polyunsaturated fatty acid deficit in patients with bipolar mania. Eur Neuropsychopharmacol.

[101] Evans S.J., Kamali M., Prossin A.R., Harrington G.J., Ellingrod V.L., McInnis M.G., Burant C.F. (2012). Association of plasma omega-3 and omega-6 lipids with burden of disease measures in bipolar subjects. J Psychiatr Res.

[102] Shakeri J., Farnia V., Valinia K., Hashemian A.H., Bajoghli H., Holsboer-Trachsler E., Brand S. (2015). The relationship between lifetime suicide attempts, serum lipid levels, and metabolic syndrome in patients with bipolar disorders. Int J Psychiatry Clin Pract.

[103] Aguglia A., Solano P., Giacomini G., Caprino M., Conigliaro C., Romano M. (2019). The association between dyslipidemia and lethality of suicide attempts: A case-control study. Front Psychiatry.

[104] Ainiyet B., Rybakowski J.K. (2014). Suicidal behaviour and lipid levels in unipolar and bipolar depression. Acta Neuropsychiatr.

[105] da Graça Cantarelli M., Nardin P., Buffon A., Eidt M.C., Antônio Godoy L., Fernandes B.S., Gonçalves C.A. (2015). Serum triglycerides, but not cholesterol or leptin, are decreased in suicide attempters with mood disorders. J Affect Disord.

[106] Vuksan-Ćusa B., Marčinko D., Nad S., Jakovljević M. (2009). Differences in cholesterol and metabolic syndrome between bipolar disorder men with and without suicide attempts. Acta Neuropsychiatr.

[107] Du Y., Dong J.H., Chen L., Liu H., Zheng G.E., Chen G.Y., Cheng Y. (2022). Metabolomic identification of serum exosome-derived biomarkers for bipolar disorder. Oxid Med Cell Longev.

[108] Guo L., Zhang T., Li R., Cui Z.Q., Du J., Yang J.B. (2022). Alterations in the plasma lipidome of adult women with bipolar disorder: A mass spectrometry-based lipidomics research. Front Psychiatry.

[109] Zhang T., Guo L., Li R., Wang F., Yang W.M., Yang J.B. (2022). Alterations of plasma lipids in adult women with major depressive disorder and bipolar depression. Front Psychiatry.

[110] Brunkhorst-Kanaan N., Klatt-Schreiner K., Hackel J., Schröter K., Trautmann S., Hahnefeld L. (2019). Targeted lipidomics reveal derangement of ceramides in major depression and bipolar disorder. Metabolism.

[111] Ribeiro H.C., Sen P., Dickens A., Santa Cruz E.C., Orešič M., Sussulini A. (2022). Metabolomic and proteomic profiling in bipolar disorder patients revealed potential molecular signatures related to hemostasis. Metabolomics.

[112] Avigdor B.E., Yang K., Shinder I., Orsburn B.C., Rais R., Kano S.I. (2021). Characterization of antipsychotic medications, amino acid signatures, and platelet-activating factor in first-episode psychosis. Biomark Neuropsychiatry.

[113] Igarashi M., Ma K., Gao F., Kim H.W., Greenstein D., Rapoport S.I., Rao J.S. (2010). Brain lipid concentrations in bipolar disorder. J Psychiatr Res.

[114] Boston P.F., Dursun S.M., Reveley M.A. (1996). Cholesterol and mental disorder. Br J Psychiatry.

[115] Steegmans P.H., Fekkes D., Hoes A.W., Bak A.A., van der Does E., Grobbee D.E. (1996). Low serum cholesterol concentration and serotonin metabolism in men. BMJ.

[116] Sarchiapone M., Camardese G., Roy A., Della Casa S., Satta M.A., Gonzalez B. (2001). Cholesterol and serotonin indices in depressed and suicidal patients. J Affect Disord.

[117] Scanlon S.M., Williams D.C., Schloss P. (2001). Membrane cholesterol modulates serotonin transporter activity. Biochemistry.

[118] Oakes V., Domene C. (2019). Influence of cholesterol and its stereoisomers on members of the serotonin receptor family. J Mol Biol.

[119] Berk M., Kapczinski F., Andreazza A.C., Dean O.M., Giorlando F., Maes M. (2011). Pathways underlying neuroprogression in bipolar disorder: Focus on inflammation, oxidative stress and neurotrophic factors. Neurosci Biobehav Rev.

[120] Soran H., Schofield J.D., Durrington P.N. (2015). Antioxidant properties of HDL. Front Pharmacol.

[121] Roshanaei-Moghaddam B., Katon W.J., Russo J. (2009). The longitudinal effects of depression on physical activity. Gen Hosp Psychiatry.

[122] Minassian A., Henry B.L., Geyer M.A., Paulus M.P., Young J.W., Perry W. (2010). The quantitative assessment of motor activity in mania and schizophrenia. J Affect Disord.

[123] De Hert M., Detraux J., van Winkel R., Yu W., Correll C.U. (2011). Metabolic and cardiovascular adverse effects associated with antipsychotic drugs. Nat Rev Endocrinol.

[124] Tschoner A., Engl J., Laimer M., Kaser S., Rettenbacher M., Fleischhacker W.W. (2007). Metabolic side effects of antipsychotic medication. Int J Clin Pract.

[125] Wenk M.R. (2010). Lipidomics: New tools and applications. Cell.

[126] Hasin Y., Seldin M., Lusis A. (2017). Multi-omics approaches to disease. Genome Biol.

[127] Topuz R.D., Gorgulu Y., Uluturk M.K. (2022). Could serum endocannabinoid and N-acylethanolamine levels be important in bipolar disorder?. World J Biol Psychiatry.

[128] Simopoulos A.P. (2002). Omega-3 fatty acids in inflammation and autoimmune diseases. J Am Coll Nutr.

[129] Rosenblat J.D., McIntyre R.S. (2016). Bipolar disorder and inflammation. Psychiatr Clin North Am.

[130] Fries G.R., Walss-Bass C., Bauer M.E., Teixeira A.L. (2019). Revisiting inflammation in bipolar disorder. Pharmacol Biochem Behav.

[131] Innes J.K., Calder P.C. (2018). Omega-6 fatty acids and inflammation. Prostaglandins Leukot Essent Fatty Acids.

[132] Farooqui A.A., Horrocks L.A., Farooqui T. (2000). Glycerophospholipids in brain: Their metabolism, incorporation into membranes, functions, and involvement in neurological disorders. Chem Phys Lipids.

[133] Hannun Y.A., Obeid L.M. (2018). Sphingolipids and their metabolism in physiology and disease. Nat Rev Mol Cell Biol.

[134] Ghosh S., Strum J.C., Bell R.M. (1997). Lipid biochemistry: Functions of glycerolipids and sphingolipids in cellular signaling. FASEB J.

[135] Züllig T., Köfeler H.C. (2021). High resolution mass spectrometry in lipidomics. Mass Spectrom Rev.

[136] Holzlechner M., Eugenin E., Prideaux B. (2019). Mass spectrometry imaging to detect lipid biomarkers and disease signatures in cancer. Cancer Rep (Hoboken).

[137] Murphy R.C. (2020). Lipid mass spectrometry: A path traveled for 50 years. J Mass Spectrom.

[138] Dixon P., Hollingworth W., Pike K., Reynolds R., Stoddart M., MacGowan A. (2021). Cost-effectiveness of rapid laboratory-based mass-spectrometry diagnosis of bloodstream infection: Evidence from the RAPIDO randomised controlled trial. BMJ (Open).

[139] Lipidomics Standards Initiative Consortium (2019). Lipidomics needs more standardization. Nat Metab.

[140] Lam S.M., Tian H., Shui G. (2017). Lipidomics, en route to accurate quantitation. Biochim Biophys Acta Mol Cell Biol Lipids.

[141] Jones H.J., Borges M.C., Carnegie R., Mongan D., Rogers P.J., Lewis S.J. (2021). Associations between plasma fatty acid concentrations and schizophrenia: A two-sample Mendelian randomisation study. Lancet Psychiatry.

[142] Bouatra S., Aziat F., Mandal R., Guo A.C., Wilson M.R., Knox C. (2013). The human urine metabolome. PLoS One.

[143] Engelborghs S., Niemantsverdriet E., Struyfs H., Blennow K., Brouns R., Comabella M. (2017). Consensus guidelines for lumbar puncture in patients with neurological diseases. Alzheimers Dement (Amst).

[144] Insel T., Cuthbert B., Garvey M., Heinssen R., Pine D.S., Quinn K. (2010). Research domain criteria (RDoC): Toward a new classification framework for research on mental disorders. Am J Psychiatry.

[145] Greenebaum S.L.A., Nierenberg A.A. (2020). More on the NIMH RDoC initiative. Bipolar Disord.

[146] Kuwano N., Kato T.A., Setoyama D., Sato-Kasai M., Shimokawa N., Hayakawa K. (2018). Tryptophan-kynurenine and lipid related metabolites as blood biomarkers for first-episode drug-naïve patients with major depressive disorder: An exploratory pilot case-control study. J Affect Disord.

[147] Sankaranarayanan A., Pratt R., Anoop A., Smith A., Espinoza D., Ramachandran P., Tirupati S. (2021). Serum lipids and suicidal risk among patients with schizophrenia spectrum disorders: Systematic review and meta-analysis. Acta Psychiatr Scand.

[148] Li H., Zhang X., Sun Q., Zou R., Li Z., Liu S. (2020). Association between serum lipid concentrations and attempted suicide in patients with major depressive disorder: A meta-analysis. PLoS One.

[149] Friedman J.N.W., Hurley R.A., Taber K.H. (2006). Bipolar disorder: Imaging state versus trait. J Neuropsychiatry Clin Neurosci.

