# Supplementary material for: Lipid Biomarker Research in Bipolar Disorder: A Scoping Review of Trends, Challenges, and Future Directions
Source: Biol Psychiatry Glob Open Sci. 2023 Jul 23;3(4):594–604. doi: 10.1016/j.bpsgos.2023.07.004 (PMC10593953; doi:10.1016/j.bpsgos.2023.07.004)
Supplement: Supplemental Data [file mmc1.pdf]

SUPPLEMENTARY INFORMATION

Lipid Biomarker Research in Bipolar Disorder: A Scoping Review of Trends, Challenges and Future Directions

Hiller *et al.*

Note S1. Overview of protocol changes.

The original literature search was developed to identify records on the role of lipids in bipolar disorder (BD). The scope of the review was then specified to focus on lipids as potential biomarkers of BD, before the protocol was published, and before the blinding was turned off in Rayyan. After the protocol was registered, we also specified three specific aims to the scoping review: to gain overview of lipid biomarker research in BD in terms of case sample sizes, to identify frequently reported study limitations, and to briefly present key research findings. The following changes were applied after online registration of the protocol:

**Protocol change**
Added exclusion criteria:
Comments and unpublished literature.
Reports that assessed biological samples which were not body fluids, such as tissue biopsies and in vivo spectroscopies.
Reports that assessed postmortem samples.
Reports that only measured molecules that are related to lipids, but are not lipids themselves, including molecules related to lipid peroxidation.
Epidemiological studies where the purpose was to measure prevalence of lipid alterations in BD populations, rather than identify biomarkers.
Reports that assessed metabolic syndrome (MetS) in BD without distinguishing between individual MetS components.
Reports that investigate associations between lipids and BD for other reasons than assessing lipids as biomarkers for BD, such as explaining the prevalence of cardiovascular disorders or MetS in BD.
Added inclusion criteria:
All individuals diagnosed with a bipolar disorder according to all ICD and DSM diagnostic classification system editions.
Added data charting items:
Longitudinal.
Lipids investigated.

Note S2. Documentation of literature search.

The following databases were searched:

Database	Number of retrieved references
**MEDLINE (Ovid)**	3297
**Embase (Ovid)**	3406
**APA PsycInfo**	965
**Scopus**	4448
**Number of references before deduplication:**	12116
**Number of duplicate references removed**	4962
**Number of references after deduplication:**	7154

All searches were performed on 16.09.2022 by academic librarian Hilde Strømme, University of Oslo Library of Medicine and Science.

Ovid MEDLINE(R) ALL <1946 to September 15, 2022>

**1**	**Lipidomics/ or exp lipids/**	**1252123**
**2**	(lipid* or ((fatty or decanoic or capric or eicosanoic or arachidic or mycophenolic or palmitic or stearic or tetrahydrolinoleic or octadecanoic or dihydrooleic or thioctic or lipoic or phosphatidic) adj acid*) or ((mycophenolate or sodium) adj mofetil) or decanoates or endocannabinoid* or lipid* ceroid* or myfortic* or palmitate* or hexadecanoate* or stearate* or octadecanoate* or "palmitoyl coenzyme a" or ((fatty or butyl or hexyl) adj alcohol*) or butanol* or hydroxybutane* or butylhydroxide* or chlorobutanol* or chlorbutol* or trichlorbutanol* or acetonchloroform or chloretone* or dolichol* or hydroxyhexane* or hexanol* or amylcarbinol* or cyclohexanol* or desvenlafaxine* or eucalyptol* or cineol* or soledum or venlafaxine or effexor or trevilor or vandral or efexor or dobupal or menthol or tramadol or glycerid* or diglycerid* or monoglycerid* or triglycerid* or triacetin* or triolein* or glycolipid* or glycosphingolipid* or sphingoglycolipid* or asialoganglioside* or glycosylphosphatidylinositol* or "Glycosyl* Phosphatidylinositol*" or "PI Glycan" or "GPI Membrane Anchor*" or lipoperoxide* or ((lipid or fatty) adj2 (peroxide* or hydroperoxid* or membrane* or bilayer*)) or lipoglycans or lipopolysaccharide* or lipoprotein* or apolipoprotein* or phosphatid* or phospholipid* or glycerolphosphate* or glycerophosphate* or (Glycerol adj2 phosphocholine*) or (choline adj (alphoscerate* or alfoscerate*)) or glycerylphosphorylcholine* or glycerophosphorylcholine* or Diacylglycerophosphate* or "ammonium phosphatidate*" or sphingolipid* or lysosphingolipid* or sterol* or oil or oils).tw,kw,kf.	1321625
**3**	Bipolar Disorder/	44157
**4**	(((bipolar or cyclothymic) adj3 (disord* or illness* or personalit* or psychos* or depress*)) or ((manic or mano) adj3 (depress* or disord*)) or maniodepress*).tw,kw,kf.	44991
**5**	(1 or 2) and (3 or 4)	3603

**6**	5 not (editorial or interview or letter or comment or congress or legal case or meeting abstract).pt.	3297

Embase Classic+Embase <1947 to 2022 September 15>

**1**	**Lipidomics/ or exp lipid/**	**1860756**
**2**	(lipid* or ((fatty or decanoic or capric or eicosanoic or arachidic or mycophenolic or palmitic or stearic or tetrahydrolinoleic or octadecanoic or dihydrooleic or thioctic or lipoic or phosphatidic) adj acid*) or ((mycophenolate or sodium) adj mofetil) or decanoates or endocannabinoid* or lipid* ceroid* or myfortic* or palmitate* or hexadecanoate* or stearate* or octadecanoate* or "palmitoyl coenzyme a" or ((fatty or butyl or hexyl) adj alcohol*) or butanol* or hydroxybutane* or butylhydroxide* or chlorobutanol* or chlorbutol* or trichlorbutanol* or acetonchloroform or chloretone* or dolichol* or hydroxyhexane* or hexanol* or amylcarbinol* or cyclohexanol* or desvenlafaxine* or eucalyptol* or cineol* or soledum or venlafaxine or effexor or trevilor or vandral or efexor or dobupal or menthol or tramadol or glycerid* or diglycerid* or monoglycerid* or triglycerid* or triacetin* or triolein* or glycolipid* or glycosphingolipid* or sphingoglycolipid* or asialoganglioside* or glycosylphosphatidylinositol* or "Glycosyl* Phosphatidylinositol*" or "PI Glycan" or "GPI Membrane Anchor*" or lipoperoxide* or ((lipid or fatty) adj2 (peroxide* or hydroperoxid* or membrane* or bilayer*)) or lipoglycans or lipopolysaccharide* or lipoprotein* or apolipoprotein* or phosphatid* or phospholipid* or glycerolphosphate* or glycerophosphate* or (Glycerol adj2 phosphocholine*) or (choline adj (alphoscerate* or alfoscerate*)) or glycerylphosphorylcholine* or glycerophosphorylcholine* or Diacylglycerophosphate* or "ammonium phosphatidate*" or sphingolipid* or lysosphingolipid* or sterol* or oil or oils).tw,kw,kf.	1702570
**3**	exp bipolar disorder/	75596
**4**	(((bipolar or cyclothymic) adj3 (disord* or illness* or personalit* or psychos* or depress*)) or ((manic or mano) adj3 (depress* or disord*)) or maniodepress*).tw,kw,kf.	67846
**5**	(1 or 2) and (3 or 4)	4791
**6**	5 not (Conference abstract or Conference paper or Conference review or Editorial or Letter or Note or Short survey).pt.	3406

APA PsycInfo <1987 to September Week 1 2022>

**1**	**Lipids/**	**3298**
**2**	(lipid* or ((fatty or decanoic or capric or eicosanoic or arachidic or mycophenolic or palmitic or stearic or tetrahydrolinoleic or octadecanoic or dihydrooleic or thioctic or lipoic or phosphatidic) adj acid*) or ((mycophenolate or sodium) adj mofetil) or decanoates or endocannabinoid* or lipid* ceroid* or myfortic* or palmitate* or hexadecanoate* or stearate* or octadecanoate* or "palmitoyl coenzyme a" or ((fatty or butyl or hexyl) adj alcohol*) or butanol* or hydroxybutane* or butylhydroxide* or chlorobutanol* or chlorbutol* or trichlorbutanol* or acetonchloroform or chloretone* or dolichol* or hydroxyhexane* or hexanol* or amylcarbinol* or cyclohexanol* or desvenlafaxine* or eucalyptol* or cineol* or soledum or venlafaxine or effexor or trevilor or vandral or efexor or dobupal or menthol or tramadol or glycerid* or diglycerid* or monoglycerid* or triglycerid* or triacetin* or triolein* or glycolipid* or glycosphingolipid* or sphingoglycolipid* or asialoganglioside* or glycosylphosphatidylinositol* or "Glycosyl* Phosphatidylinositol*" or "PI Glycan" or "GPI Membrane Anchor*" or lipoperoxide* or ((lipid or fatty) adj2 (peroxide* or	38825

	hydroperoxid* or membrane* or bilayer*)) or lipoglycans or lipopolysaccharide* or lipoprotein* or apolipoprotein* or phosphatid* or phospholipid* or glycerolphosphate* or glycerophosphate* or (Glycerol adj2 phosphocholine*) or (choline adj (alphoscerate* or alfoscerate*)) or glycerylphosphorylcholine* or glycerophosphorylcholine* or Diacylglycerophosphate* or "ammonium phosphatidate*" or sphingolipid* or lysosphingolipid* or sterol* or oil or oils).tw.	
**3**	exp Bipolar Disorder/	30700
**4**	(((bipolar or cyclothymic) adj3 (disord* or illness* or personalit* or psychos* or depress*)) or ((manic or mano) adj3 (depress* or disord*)) or maniodepress*).tw.	38878
**5**	(1 or 2) and (3 or 4)	1019
**6**	5 not (Book or Authored Book or Edited Book or Dissertation Abstract).pt.	965

Scopus

TITLE-ABS-KEY (((lipid* OR ("fatty" OR "decanoic" OR "capric" OR "eicosanoic" OR "arachidic" OR "mycophenolic" OR "palmitic" OR "stearic" OR "tetrahydrolinoleic" OR "octadecanoic" OR "dihydrooleic" OR "thioctic" OR "lipoic" OR "phosphatidic") W/0 "acid*") OR (("mycophenolate" OR "sodium") W/0 "mofetil") OR "decanoates" OR "endocannabinoid*" OR "lipid* ceroid*" OR "myfortic*" OR "palmitate*" OR "hexadecanoate*" OR "stearate*" OR "octadecanoate*" OR "palmitoyl coenzyme a" OR (("fatty" OR "butyl" OR "hexyl") W/0 "alcohol*") OR "butanol*" OR "hydroxybutane*" OR "butylhydroxide*" OR "chlorobutanol*" OR "chlorbutol*" OR "trichlorbutanol*" OR "acetonchloroform" OR "chloretone*" OR "dolichol*" OR "hydroxyhexane*" OR "hexanol*" OR "amylcarbinol*" OR "cyclohexanol*" OR "desvenlafaxine*" OR "eucalyptol*" OR "cineol*" OR "soledum" OR "venlafaxine" OR "effexor" OR "trevilor" OR "vandral" OR "efexor" OR "dobupal" OR "menthol" OR "tramadol" OR "glycerid*" OR "diglycerid*" OR "monoglycerid*" OR "triglycerid*" OR "triacetin*" OR "triolein*" OR "glycolipid*" OR "glycosphingolipid*" OR "sphingoglycolipid*" OR "asialoganglioside*" OR "glycosylphosphatidylinositol*" OR "Glycosyl* Phosphatidylinositol*" OR "PI Glycan" OR "GPI Membrane Anchor*" OR "lipoperoxide*" OR (("lipid" OR "fatty") W/1 ("peroxide*" OR "hydroperoxid*" OR "membrane*" OR "bilayer*")) OR "lipoglycans" OR "lipopolysaccharide*" OR "lipoprotein*" OR "apolipoprotein*" OR "phosphatid*" OR "phospholipid*" OR "glycerolphosphate*" OR "glycerophosphate*" OR ("Glycerol" W/1 "phosphocholine*") OR ("choline" W/0 ("alphoscerate*" OR "alfoscerate*")) OR "glycerylphosphorylcholine*" OR "glycerophosphorylcholine*" OR "Diacylglycerophosphate*" OR "ammonium phosphatidate*" OR "sphingolipid*" OR "lysosphingolipid*" OR "sterol*" OR "oil" OR "oils") AND ((("bipolar" OR "cyclothymic") W/2 ("disord*" OR "illness*" OR "personalit*" OR "psychos*" OR "depress*")) OR (("manic" OR "mano") W/2 ("depress*" OR "disord*")) OR "maniodepress*")) AND (EXCLUDE (DOCTYPE , "le") OR EXCLUDE (DOCTYPE , "cp") OR EXCLUDE (DOCTYPE , "sh") OR EXCLUDE (DOCTYPE , "ed") OR EXCLUDE (DOCTYPE , "ch") OR EXCLUDE (DOCTYPE , "bk") OR EXCLUDE (DOCTYPE , "tb"))

4448 hits

Note S3. Lipids investigated in lipidomic studies

In targeted lipidomics (25 studies, Table S3), 15 studies investigated polyunsaturated fatty acids (PUFA) (such as eicosapentaenoic acid (EPA), docosahexaenoic acid (DHA) and (eicosadienoic acid (EDA)) in erythrocyte membranes or as serum free fatty acids. Global lipidomics or metabolomics were conducted in nine reports, of which seven analyzed blood samples, and some additional studies reported on specific lipids such as phospholipids, cortisol, very low-density lipoprotein (VLDL), nervonic acid and endocannabinoids.

Note S4. Reported limitations.

The most frequently reported weakness of the studies was small sample size (51 reports). Study designs were commonly reported as suboptimal, with the focus on cross-sectional design without the ability to investigate causal relationship and traits over time (37 reports). In addition, authors report that potential confounders were not accounted for, such as physical exercise and diet of the subjects, smoking status, socioeconomic status, comorbidities and other psychosocial factors (39 reports). A large proportion did not account for the patients' drug regimens (34 reports).

Note S5. Detailed description of study results.

Case-control comparisons. In the reviewed literature, nine of the papers that utilized standard laboratory assessments found significantly decreased TC in bipolar patients compared to controls (1–9), 19 found no significant difference (10–28), and four found significantly increased TC in bipolar patients compared to controls (29–32). Regarding TG, one publication found significantly decreased TG in BD compared to controls (5), 16 found no significant difference (7,8,10–13,15,16,18,22,23,25,26,28,29,33), while twelve publications found significant increases of TG in patients with BD compared to controls (3,4,6,9,17,19–21,24,27,31,34). LDL was found to be significantly decreased in bipolar patients compared to controls in five papers (4,5,7–9), while 19 (3,6,10–19,21–25,27,28) and two (29,31) publications found no significant difference between the groups and significantly increased LDL in cases compared to controls, respectively. Nine publications reported significantly decreased HDL in cases (3,4,6,9,19,21,23,24,34), 26 found no difference between cases and controls (2,5,7,8,10–18,20,22,25–29,31,33,35–38), and none reported any significant increase in HDL between the groups.

Depressive episode. Three publications found decreased TC to be significantly associated with depressive episodes in BD (2,9,39), while 20 papers found no significant differences in TC level between depressed and non-depressed BD (6,8,10,11,15,19–24,26–29,40–44). Four papers found significant associations between increased TC and depressive episodes in BD (12,13,30,45). Only one paper found decreased TG to be significantly associated with depressive episodes (39), 19 found no significant difference (6,8,10,12,13,15,19–23,26–29,40,41,43,45), and four papers found increased TG to be significantly associated with depressive episodes (9,11,24,44). One (9) and two (9,24) papers found decreased LDL and HDL, respectively, to be significantly associated with depressive episodes in BD, 17 (6,8,10–12,15,19,21–24,27–29,39–41) and 24 (2,6,8,10,11,13,15,19–23,26–29,36–41,44,46) found no significant differences in LDL and HDL between depressed and non-depressed, while one paper found significant associations between increased LDL and depressive episodes (13), and one paper found increased HDL to be significantly associated with depressive episodes in BD (12).

Manic episode. Nine papers found significantly decreased TC in manic BD patients (1,2,7,9,12,15,32,41,47), 15 found no significant difference between manic and non-manic patients (6,14,19,20,22–24,26,27,29,30,40,42,45,48), and no papers found any significant

associations between increased TC and mania. One paper found significantly decreased TG in mania (15), 15 found no significant change in TG level associated with mania (6,7,12,19,20,22,23,26,27,29,33,40,41,45,48), and two papers found increased TG to be associated with mania in BD (9,24). Regarding LDL, four publications found significant associations between decreased LDL and mania in BD (7,9,12,41), and twelve papers found no significant decrease or increase (6,14,15,19,22–24,27,29,40,45,48). Three papers found HDL significantly decreased in manic BD patients (9,12,24), while 20 papers found no significant change in HDL level associated with mania (2,6,7,14,15,19,20,22,23,26,27,29,33,36,38,40,41,45,46,48). No papers found any significant increases in LDL or HDL in regards to mania in patients with BD.

Suicidality. Three papers found decreased levels of TC to be significantly associated with suicidal tendencies (10,49,50), eight papers no difference between suicidality and non-suicidality (40,42,51–56), and two papers found significantly increased TC in bipolar patients with suicidal tendencies (8,57). Four papers found TG to be significantly decreased in suicidal patients (10,49,50,53), eight found no significant changes (8,40,51,52,54–57), and no publications reported significant increases in TG associated with suicidality in BD. Two papers reported significantly decreased LDL in suicidal patients (10,49), while seven reported no significant difference (8,40,50,51,53,56,57) and none reported significantly increased LDL. One report showed significantly decreased HDL in suicidality (49), eleven reports showed no significant changes in HDL levels (10,36–38,40,50,52–54,56,57), and one report showed significantly increased HDL levels associated with suicidality in BD (8).

Table S1. Studies excluded following full-text review.

Reference	Report	Reason for exclusion
(58)	Abdulla et al. 1975	Published before 1990
(59)	Adibhatla et al. 2006	Background article
(60)	Adibhatla et al. 2007	Wrong study design
(61)	Adibhatla et al. 2008	Wrong study design
(62)	Akarsu et al. 2018	Wrong outcome
(63)	Akimoto 2004	Foreign language
(64)	Akyol et al. 2020	Wrong study design
(65)	Alam et al. 2017	Wrong study design
(66)	Alarcon et al. 1985	Published before 1990
(67)	Ande et al. 2019	Wrong study design
(68)	Andreassen et al. 2015	Wrong outcome
(69)	Arnsten et al. 2008	Wrong study design
(70)	Astorg 2007	Wrong study design
(71)	Atagun et al. 2018	Wrong outcome
(72)	Bai et al. 2020	Wrong outcome
(73)	Baptista et al. 2015	Wrong outcome
(74)	Bartoli et al. 2017	Wrong study design
(75)	Bavaresco et al. 2020	Wrong study design
(76)	Beasley et al. 2005	Wrong outcome
(77)	Beasley et al. 2020	Wrong outcome
(78)	Bellivier et al. 1997	Wrong method
(79)	Bennett et al. 2000	Wrong study design
(80)	Bly et al. 2014	Wrong method
(81)	Bocchetta et al. 2001	Wrong method
(82)	Bonifacio et al. 2017	Wrong outcome
(83)	Bonifacio et al. 2021	Wrong outcome
(84)	Bortolasci et al. 2014	Wrong outcome
(85)	Bortolasci et al. 2015	Wrong method
(86)	Brandrup et al. 1967	Published before 1990
(87)	Bristot et al. 2020	Wrong method
(88)	Brown et al. 1993	Wrong study design
(89)	Brown et al. 2014	Wrong study design
(90)	Burghardt et al. 2013	Wrong outcome
(91)	Campbell et al. 2019	Wrong study design
(92)	Ceylan et al. 2018	Wrong method
(93)	Ceylan et al. 2020	Wrong outcome
(94)	Chang et al. 2017	Wrong method
(95)	Chiurchiu et al. 2016	Wrong study design
(96)	Chowdhury et al. 2017	Wrong outcome
(97)	Clark et al. 2016	Wrong population
(98)	Clayton et al. 2013	Wrong study design
(99)	Cocchi et al. 2012	Wrong outcome
(100)	Cocchi et al. 2014	Wrong study design
(101)	Cocchi et al. 2017	Wrong study design

(102)	Coryell et al. 2007	Wrong population
(103)	Cudney et al. 2014	Wrong method
(104)	Cuellar-Barboza et al. 2021	Wrong outcome
(105)	Cuturic et al. 2016	Wrong outcome
(106)	Cyrino et al. 2021	Wrong study design
(107)	Dalkner et al. 2018	Wrong outcome
(108)	Davison et al. 2012	Wrong method
(109)	De Berardis et al. 2009	Wrong study design
(110)	Dean et al. 2006	Wrong study design
(111)	Dean et al. 2008	Wrong outcome
(112)	Dieset et al. 2019	Wrong method
(113)	Dolapoglu et al. 2021	Wrong outcome
(114)	Dumas et al. 2013	Wrong study design
(115)	Elvåshagen et al. 2016	Wrong outcome
(116)	Emanuele et al. 2006	Wrong outcome
(117)	Erkiran et al. 2002	Foreign language
(118)	Ersan et al. 2021	Wrong outcome
(119)	Ertekin et al. 2018	Wrong outcome
(120)	Ezzaher et al. 2010	Foreign language
(121)	Ezzaher et al. 2011	Wrong outcome
(122)	Fagiolini et al. 2008	Wrong study design
(123)	Farstad 1966	Published before 1990
(124)	Fe'li et al. 2020	Wrong outcome
(125)	Fern et al. 2020	Wrong method
(126)	Fiedorowicz et al. 2007	Not used ICD/DSM
(127)	Fiedorowicz et al. 2010	Not used ICD/DSM
(128)	Fleet-Michaliszyn et al. 2008	Wrong outcome
(129)	Foguet-Boreu et al. 2020	Wrong outcome
(130)	Freeman et al. 2011	Wrong study design
(131)	Frey et al. 2007	Wrong method
(132)	Frey et al. 2007	Wrong outcome
(132)	Frey et al. 2007	Wrong outcome (duplicate)
(133)	Fries et al. 2017	Wrong method
(134)	Geoffroy et al. 2017	Wrong outcome
(135)	Ghosh et al. 2017	Wrong method
(136)	Glen et al. 1996	Wrong population
(137)	Godin et al. 2017	Wrong outcome
(138)	Golkowski et al. 2018	Wrong method
(139)	Gubert et al. 2013	Wrong method
(140)	Hamazaki et al. 2010	Wrong method
(141)	Hamazaki et al. 2012	Wrong method
(142)	Hamazaki et al. 2013	Wrong method
(143)	Hamazaki et al. 2013	Wrong method
(144)	Hamazaki et al. 2015	Wrong method
(145)	Hamazaki et al. 2017	Wrong method
(146)	Hammond et al. 2007	Wrong study design
(147)	Hashimoto 2018	Wrong study design
(148)	Hatch et al. 2015	Wrong method

(149)	Horrobin 1981	Published before 1990
(150)	Horrobin et al. 1999	Wrong study design
(151)	Horrobin et al. 1999	Wrong study design
(152)	Howells et al. 2013	Wrong method
(153)	Hu et al. 2019	Wrong method
(154)	Hullin et al. 1970	Published before 1990
(155)	Hylen et al. 2021	Wrong population
(156)	Igarashi et al. 2010	Wrong method
(157)	Ikenaga et al. 2015	Wrong outcome
(158)	Jakovljevic et al. 2007	Wrong study design
(159)	Joshi et al. 2014	Wrong study design
(160)	Kalelioglu et al. 2018	Wrong outcome
(161)	Kapczinski et al. 2008	Wrong method
(162)	Kavoor et al. 2015	Wrong study design
(163)	Kenna et al. 2009	Wrong study design
(164)	Kenneson et al. 2017	Wrong outcome
(165)	Kesebir et al. 2017	Wrong outcome
(166)	Kilic et al. 2020	Wrong method
(167)	Kim et al. 2009	Wrong outcome
(168)	Kim et al. 2016	Wrong outcome
(169)	Knöchel et al. 2017	Wrong outcome
(170)	Knowles et al. 2017	Wrong method
(171)	Kovaleva et al. 1988	Published before 1990
(172)	Kulak-Bejda et al. 2021	Wrong study design
(173)	Kuloglu et al. 2002	Wrong method
(174)	Kurt et al. 2009	Foreign language
(175)	Kusumi et al. 2018	Wrong outcome
(176)	Lang et al. 2019	Wrong study design
(177)	Lange 2020	Wrong study design
(178)	Le-Niculescu et al. 2011	Wrong population
(179)	Lee et al. 2021	Wrong outcome
(180)	Lengvenyte et al. 2022	Wrong outcome
(181)	Levey et al. 2016	Wrong outcome
(182)	Li et al. 2022	Wrong population
(183)	Liao et al. 2012	Not used ICD/DSM
(184)	Liu et al. 2015	Wrong study design
(185)	Lopuszanska et al. 2017	Wrong outcome
(186)	Lunding et al. 2021	Wrong outcome
(187)	Maes et al. 2019	Wrong study design
(188)	Maida et al. 2006	Wrong outcome
(189)	Mallick et al. 2019	Wrong study design
(190)	Mansur et al. 2016	Wrong outcome
(191)	Margari et al. 2013	Wrong outcome
(192)	Mcnamara et al. 2006	Wrong method
(193)	Mcnamara et al. 2010	Not used ICD/DSM
(194)	Mcnamara et al. 2012	Wrong study design
(195)	Mcnamara 2013	Wrong study design
(196)	Mcnamara et al. 2014	Wrong method

(197)	Mcnamara 2015	Wrong study design
(198)	Meira-Lima et al. 2003	Foreign language
(199)	Messamore et al. 2016	Wrong study design
(200)	Messamore et al. 2017	Wrong study design
(201)	Mico et al. 2011	Wrong method
(202)	Moolamalla et al. 2020	Wrong outcome
(203)	Morris et al. 2019	Wrong study design
(204)	Morris et al. 2021	Wrong study design
(205)	Muskiet et al. 2006	Wrong study design
(206)	Nasca et al. 2021	Wrong population
(207)	Newton et al. 2017	Wrong method
(208)	Omileke et al. 2020	Wrong outcome
(209)	Ong et al. 2015	Wrong study design
(210)	Ozcan et al. 2004	Wrong method
(211)	Patrick et al. 2015	Wrong study design
(212)	Pedrini et al. 2012	Wrong population
(213)	Persons et al. 2012	Wrong study design
(214)	Plana et al. 2010	Not used ICD/DSM
(215)	Quintero et al. 2019	Wrong study design
(216)	Reponen et al. 2020	Wrong outcome
(217)	Roedding et al. 2006	Wrong method
(218)	Ross et al. 2006	Wrong outcome
(219)	Rosso et al. 2015	Wrong outcome
(220)	Russell et al. 2015	Wrong outcome
(221)	Rutkofsky et al. 2017	Wrong study design
(222)	Rybakowski et al. 1996	Foreign language
(223)	Sagud et al. 2007	Background article
(224)	Saugstad 2001	Wrong study design
(225)	Saugstad 2002	Wrong study design
(226)	Saugstad 2002	Wrong study design
(227)	Saunders et al. 2016	Wrong study design
(228)	Schmechel 2007	Wrong study design
(229)	Schneider et al. 2017	Wrong study design
(230)	Schwarz et al. 2008	Wrong population
(231)	Scola et al. 2014	Wrong study design
(232)	Scola et al. 2016	Wrong method
(233)	Setareh et al. 2018	Foreign language
(234)	Sethi et al. 2017	Duplicate
(235)	Shah et al. 2019	Wrong study design
(236)	Shaheen et al. 1971	Published before 1990
(237)	Shamim et al. 2018	Wrong study design
(238)	Shapiro et al. 2022	Not used ICD/DSM
(239)	Smythies et al. 1986	Published before 1990
(240)	So et al. 2019	Wrong outcome
(241)	Sobczak et al. 2004	Wrong population
(242)	Sokol et al. 2007	Wrong outcome
(243)	Sonnik et al. 1987	Published before 1990
(244)	Soreca et al. 2012	Wrong outcome

(245)	Stemmle et al. 2009	Wrong study design
(246)	Stenberg 1929	Published before 1990
(247)	Sundram 2006	Wrong study design
(248)	Sussulini et al. 2009	Not used ICD/DSM
(249)	Swardfager et al. 2018	Wrong study design
(250)	Swartz 1990	Wrong outcome
(251)	Tatebayashi et al. 2012	Wrong outcome
(252)	Theodoropoulou et al. 2019	Foreign language
(253)	Thomas et al. 2001	Wrong outcome
(254)	Thomas et al. 2003	Wrong study design
(255)	Thomas et al. 2003	Wrong outcome
(256)	Tuncel et al. 2015	Wrong method
(257)	Tuncel et al. 2018	Wrong outcome
(258)	Turecki et al. 1998	Wrong outcome
(259)	Upton 2006	Wrong study design
(260)	Van Winkel et al. 2008	Wrong study design
(261)	Vargas et al. 2013	Not used ICD/DSM
(262)	Vemuri et al. 2011	Wrong outcome
(263)	Versace et al. 2014	Wrong method
(264)	Vila-Rodriguez et al. 2011	Wrong outcome
(265)	Vuksan-Cusa et al. 2009	Duplicate
(266)	Wiener et al. 2014	Wrong method
(267)	Wollenhaupt-Aguiar et al. 2020	Wrong method
(268)	Woo et al. 2017	Wrong outcome
(269)	Wysokinski et al. 2015	Wrong outcome
(270)	Yang et al. 2020	Wrong outcome
(271)	Yao et al. 2005	Wrong study design
(272)	Yui et al. 2016	Wrong study design
(273)	Zak et al. 2016	Foreign language
(274)	Zaki et al. 2014	Wrong outcome
(275)	Zeng et al. 2022	Not published

Table S2. Data extracted from included studies.

Data Point	Explanation
Author	Name of author(s).
Publication year	Year of study publication.
Title	Title of the study.
Country	Country(-ies) where the study was conducted.
Study design	Details about study design.
Longitudinal	Were there follow-up measurements?
Aims/purpose	The objectives of the study.
Setting	The type of clinical setting (e.g., hospital, psychiatric center etc.)
Study population	Numbers and diagnoses of patients and controls.
Phenotype(s) investigated	Symptomatology of individuals included.
Methodology	Details about the methods used for measurement of lipid levels. Critical appraisal of study method was not performed.
Biological samples	Type of sample that was collected for lipid measurement.
Lipid(s) investigated	Lipid species measured in the study.
Most relevant findings	Findings/outcomes relevant to review questions.
Authors' interpretation of outcomes	Arguments and hypotheses related to the outcomes provided by the authors.
Reported limitations	Challenges and limitations of the study reported by the authors.

See separate Excel file for Table S3.

Table S4. Frequency of reported limitations.

Reported Limitations	Number of Reports
Small sample size	51
Some potential confounders were not considered, i.e. physical acitivity, diet, smoking stuatus, sosioeconomic status, pshychosocial factors…	39
Cross-sectional design of study	37
Patients were taking medication	34
Retrospective design of study	15
No control group	12
No limitations reported	7
Not longitudinal study	5

Supplementary References

1. Atmaca M, Kuloglu M, Tezcan E, Ustundag B, Bayik Y. Serum leptin and cholesterol levels in patients with bipolar disorder. Neuropsychobiology. 2002;46(4):176–9.

2. De Berardis D, Conti CM, Campanella D, Carano A, Scali M, Valchera A, et al. Evaluation of C-reactive protein and total serum cholesterol in adult patients with bipolar disorder. Int J Immunopathol Pharmacol. 2008 Jun;21(2):319–24.

3. Furtjes AE, Coleman JRI, Tyrrell J, Lewis CM, Hagenaars SP. Associations and limited shared genetic aetiology between bipolar disorder and cardiometabolic traits in the UK Biobank. Psychol Med. :1–10.

4. Glueck CJ, Tieger M, Kunkel R, Hamer T, Tracy T, Speirs J. Hypocholesterolemia and affective disorders. Am J Med Sci. 308(4):218–25.

5. Guidara W, Messedi M, Maalej M, Naifar M, Khrouf W, Grayaa S, et al. Plasma oxysterols: Altered level of plasma 24-hydroxycholesterol in patients with bipolar disorder. J Steroid Biochem Mol Biol. 2021 Jul 1;211:105902.

6. Qiu Y, Li S, Teng Z, Tan Y, Xu X, Yang M, et al. Association between abnormal glycolipid level and cognitive dysfunction in drug-naive patients with bipolar disorder. J Affect Disord. 2022 Sep;297:477–85.

7. Sagud M, Mihaljevic-Peles A, Pivac N, Jakovljevic M, Muck-Seler D. Platelet serotonin and serum lipids in psychotic mania. J Affect Disord. 97(1):247–51.

8. Su M, Li E, Tang C, Zhao Y, Liu R, Gao K. Comparison of blood lipid profile/thyroid function markers between unipolar and bipolar depressed patients and in depressed patients with anhedonia or suicidal thoughts. Mol Med. 25(1):51.

9. Wei Y, Wang T, Li G, Feng J, Deng L, Xu H, et al. Investigation of systemic immune-inflammation index, neutrophil/high-density lipoprotein ratio, lymphocyte/high-density lipoprotein ratio, and monocyte/high-density lipoprotein ratio as indicators of inflammation in patients with schizophrenia and bipolar disorder. Front Psychiatry Front Res Found. 2022;13:941728.

10. Ainiyet B, Rybakowski JK. Suicidal behaviour and lipid levels in unipolar and bipolar depression. Acta Neuropsychiatr. 26(5):315–20.

11. Congio AC, Rossaneis AC, Verri Jr WA, Urbano MR, Nunes SOV. Childhood trauma, interleukin-17, C-reactive protein, metabolism, and psychosocial functioning in bipolar depression. J Affect Disord Rep [Internet]. 2022;9. Available from: https://www.scopus.com/inward/record.uri?eid=2-s2.0-85131104491&doi=10.1016%2fj.jadr.2022.100357&partnerID=40&md5=37a0f584eb658fefad319b1b9d9b0147

12. Fusar-Poli L, Amerio A, Cimpoesu P, Natale A, Salvi V, Zappa G, et al. Lipid and Glycemic Profiles in Patients with Bipolar Disorder: Cholesterol Levels Are Reduced in Mania. Med Kaunas Lith. 2020 Dec 30;57(1):28.

13. Gohar SM, Dieset I, Steen NE, Morch RH, Iversen TS, Steen VM, et al. Association between serum lipid levels, osteoprotegerin and depressive symptomatology in psychotic disorders. Eur Arch Psychiatry Clin Neurosci. 269(7):795–802.

14. Hjell G, Morch-Johnsen L, Holst R, Tesli N, Bell C, Lunding SH, et al. Disentangling the relationship between cholesterol, aggression, and impulsivity in severe mental disorders. Brain Behav. 10(9):e01751.

15. Huang YJ, Tsai SY, Chung KH, Chen PH, Huang SH, Kuo CJ. State-dependent alterations of lipid profiles in patients with bipolar disorder. Int J Psychiatry Med. 2018 Jul;53(4):273–81.

16. John S, Dharwadkar K, Motagi MV. Study on association between lipid profile values and psychiatric disorders. J Clin Diagn Res JCDR. 8(12):WC04-06.

17. Kasak M, Ceylan MF, Hesapcioglu ST, Senat A, Erel O. Peroxisome Proliferator-Activated Receptor Gamma (PPARgamma) Levels in Adolescent with Bipolar Disorder and Their Relationship with Metabolic Parameters. J Mol Neurosci. 2022 Jun;72(6):1313–21.

18. Li S, Teng Z, Qiu Y, Pan P, Wu C, Jin K, et al. Dissociation Pattern in Default-Mode Network Homogeneity in Drug-Naive Bipolar Disorder. Front Psychiatry Front Res Found. 2021;12:699292.

19. Naiberg MR, Newton DF, Collins JE, Bowie CR, Goldstein BI. Impulsivity is associated with blood pressure and waist circumference among adolescents with bipolar disorder. J Psychiatr Res. 83:230–9.

20. Naiberg MR, Newton DF, Collins JE, Dickstein DP, Bowie CR, Goldstein BI. Elevated triglycerides are associated with decreased executive function among adolescents with bipolar disorder. Acta Psychiatr Scand. 134(3):241–8.

21. Nunes SO, Piccoli de Melo LG, Pizzo de Castro MR, Barbosa DS, Vargas HO, Berk M, et al. Atherogenic index of plasma and atherogenic coefficient are increased in major depression and bipolar disorder, especially when comorbid with tobacco use disorder. J Affect Disord. 172:55–62.

22. Pan P, Qiu Y, Teng Z, Li S, Huang J, Xiang H, et al. Increased Global-Brain Functional Connectivity Is Associated with Dyslipidemia and Cognitive Impairment in First-Episode, Drug-Naive Patients with Bipolar Disorder. Neural Plast. 2021;2021:5560453.

23. Porcu M, Urbano MR, Verri WA, Machado RCR, Vargas HO, Nunes SOV. Comparison of the severity of depressive and anxiety symptoms, biomarkers, and childhood trauma among bipolar smokers and non-smokers, and controls. J Affect Disord Rep [Internet]. 2022;8. Available from: https://www.scopus.com/inward/record.uri?eid=2-s2.0-85127130312&doi=10.1016%2fj.jadr.2022.100336&partnerID=40&md5=3b0ce7c36938dede6c5ed10110263f6b

24. Sagud M, Mihaljevic-Peles A, Pivac N, Jakovljevic M, Muck-Seler D. Lipid levels in female patients with affective disorders. Psychiatry Res. 168(3):218–21.

25. Szabo A, O'Connell KS, Uel, T., Sheikh MA, Agartz I, et al. Increased circulating IL-18 levels in severe mental disorders indicate systemic inflammasome activation. Brain Behav Immun. 2022 Sep;99:299–306.

26. Van Rheenen TE, McIntyre RS, Balanza-Martinez V, Berk M, Rossell SL. Cumulative Cardiovascular Disease Risk and Triglycerides Differentially Relate to Subdomains of Executive Function in Bipolar Disorder; preliminary findings. J Affect Disord. 278:556–62.

27. Wulsin LR, Blom TJ, Durling M, Welge JA, DelBello MP, Adler CM, et al. Cardiometabolic risks and omega-3 index in recent-onset bipolar I disorder. Bipolar Disord. 20(7):658–65.

28. Zhang SF, Chen HM, Xiong JN, Liu J, Xiong J, Xie JZ, et al. Comparison of cognitive impairments with lipid profiles and inflammatory biomarkers in unipolar and bipolar depression. J Psychiatr Res. 2022 Sep;150:300–6.

29. Ezzaher A, Mouhamed DH, Mechri A, Araoud M, Neffati F, Douki W, et al. Lower paraoxonase 1 activity in Tunisian bipolar I patients. Ann Gen Psychiatry. 9:36.

30. Ghaemi SN, Shields GS, Hegarty JD, Goodwin FK. Cholesterol levels in mood disorders: high or low? Bipolar Disord. 2000 Mar;2(1):60–4.

31. Kennedy KG, Islam AH, Grigorian A, Fiksenbaum L, Mitchell RHB, McCrindle BW, et al. Elevated lipids are associated with reduced regional brain structure in youth with bipolar disorder. Acta Psychiatr Scand. 143(6):513–25.

32. Pae CU, Kim JJ, Lee SJ, Lee C, Paik IH, Lee CU. Aberration of cholesterol level in first-onset bipolar I patients. J Affect Disord. 2004 Nov 1;83(1):79–82.

33. Sanchez-Orti JV, Balanza-Martinez V, Correa-Ghisays P, Selva-Vera G, Vila-Frances J, Magdalena-Benedito R, et al. Specific metabolic syndrome components predict cognition and social functioning in people with type 2 diabetes mellitus and severe mental disorders. Acta Psychiatr Scand. 2022 Sep;146(3):215–26.

34. Dalkner N, Bengesser SA, Birner A, Fellendorf FT, Fleischmann E, Grosschadl K, et al. Metabolic Syndrome Impairs Executive Function in Bipolar Disorder. Front Neurosci. 2021;15:717824.

35. Hui L, Yin XL, Chen J, Yin XY, Zhu HL, Li J, et al. Association between decreased HDL levels and cognitive deficits in patients with bipolar disorder: a pilot study. Int J Bipolar Disord. 7(1):25.

36. Maes M, Moraes JB, Congio A, Bonifacio KL, Barbosa DS, Vargas HO, et al. Development of a Novel Staging Model for Affective Disorders Using Partial Least Squares Bootstrapping: Effects of Lipid-Associated Antioxidant Defenses and Neuro-Oxidative Stress. Mol Neurobiol. 56(9):6626–44.

37. Maes M, Congio A, Moraes JB, Bonifacio KL, Barbosa DS, Vargas HO, et al. Early Life Trauma Predicts Affective Phenomenology and the Effects are Partly Mediated by Staging Coupled with Lowered Lipid-Associated Antioxidant Defences. Biomol Concepts. 9(1):115–30.

38. Moraes JB, Maes M, Roomruangwong C, Bonifacio KL, Barbosa DS, Vargas HO, et al. In major affective disorders, early life trauma predict increased nitro-oxidative stress, lipid peroxidation and protein oxidation and recurrence of major affective disorders, suicidal behaviors and a lowered quality of life. Metab Brain Dis. 33(4):1081–96.

39. Birdsall JW, Schmitz SL, Abosi OJ, DuBose LE, Pierce GL, Fiedorowicz JG. Inflammatory and vascular correlates of mood change over 8 weeks. Heart Mind. 3(2):47–54.

40. Ahmadpanah M, Haghighi M, Jahangard L, Borzoei S, Heshmati S, Bajoghli H, et al. No evidence for metabolic syndrome and lipid profile differences in patients suffering from bipolar I disorder with and without suicide attempts. Int J Psychiatry Clin Pract. 2015;19(3):168–73.

41. Erzin G, Aydemir MC, Yuksel RN, Tatlidil Yaylaci E, Cakir B, Sezer S, et al. Serum 15-d-PGJ2 and PPARgamma levels are reduced in manic episode of bipolar disorder while IL-4 levels are not affected. Psychiatry Clin Psychopharmacol. 2019;29(3):298–306.

42. Fritze J, Schneider B, Lanczik M. Autoaggressive behaviour and cholesterol. Neuropsychobiology. 1992;26(4):180–1.

43. Mucci F, Marazziti D, Della Vecchia A, Baroni S, Massimetti G, Morana P, et al. Inflammatory and metabolic markers in patients with mood disorders. World J Biol Psychiatry. 2021;22:228–35.

44. Richter N, Juckel G, Assion HJ. Metabolic syndrome: a follow-up study of acute depressive inpatients. Eur Arch Psychiatry Clin Neurosci. 260(1):41–9.

45. Chung KH, Tsai SY, Lee HC. Mood symptoms and serum lipids in acute phase of bipolar disorder in Taiwan. Psychiatry Clin Neurosci. 61(4):428–33.

46. Kulacaoglu F, Yildirim YE, Aslan M, Izci F. Neutrophil to lymphocyte and monocyte to high-density lipoprotein ratios are promising inflammatory indicators of bipolar disorder. Nord J Psychiatry. 2022 Sep;1–6.

47. Cassidy F, Carroll BJ. Hypocholesterolemia during mixed manic episodes. Eur Arch Psychiatry Clin Neurosci. 252(3):110–4.

48. Bulbul F, Eryigit AG, Erbagci AB, Selek S, Savas H. Alterations of Lipid-Lipoprotein and Leptin in Bipolar Disorder Associated with Clinic Process. Noropsikiyatri Arsivi. 51(1):52–6.

49. Aguglia A, Solano P, Giacomini G, Caprino M, Conigliaro C, Romano M, et al. The Association Between Dyslipidemia and Lethality of Suicide Attempts: A Case-Control Study. Front Psychiatry Front Res Found. 2019;10:70.

50. Vuksan-Ćusa B, Marčinko D, Nad S, Jakovljević M. Differences in cholesterol and metabolic syndrome between bipolar disorder men with and without suicide attempts. Acta Neuropsychiatr. 2009 Jun;21 Suppl 2:66–9.

51. Capuzzi E, Bartoli F, Crocamo C, Malerba MR, Clerici M, Carra G. Recent suicide attempts and serum lipid profile in subjects with mental disorders: A cross-sectional study. Psychiatry Res. 270:611–5.

52. D'Ambrosio V, Salvi V, Bogetto F, Maina G. Serum lipids, metabolic syndrome and lifetime suicide attempts in patients with bipolar disorder. Prog Neuropsychopharmacol Biol Psychiatry. 37(1):136–40.

53. da Graca Cantarelli M, Nardin P, Buffon A, Eidt MC, Antonio Godoy L, Fern, et al. Serum triglycerides, but not cholesterol or leptin, are decreased in suicide attempters with mood disorders. J Affect Disord. 172:403–9.

54. Park S, Yi KK, Na R, Lim A, Hong JP. No association between serum cholesterol and death by suicide in patients with schizophrenia, bipolar affective disorder, or major depressive disorder. Behav Brain Funct Electron Resour BBF. 9:45.

55. Pompili M, Innamorati M, Lester D, Girardi P, Tatarelli R. Nearly lethal resuscitated suicide attempters have no low serum levels of cholesterol and triglycerides. Psychol Rep. 106(3):785–90.

56. Stenzel C, Dalkner N, Unterrainer HF, Birner A, Bengesser SA, Fellendorf FT, et al. Effects of metabolic syndrome and obesity on suicidality in individuals with bipolar disorder. J Affect Disord. 2022 Aug 15;311:1–7.

57. Shakeri J, Farnia V, Valinia K, Hashemian AH, Bajoghli H, Holsboer-Trachsler E, et al. The relationship between lifetime suicide attempts, serum lipid levels, and metabolic syndrome in patients with bipolar disorders. Int J Psychiatry Clin Pract. 19(2):124–31.

58. Abdulla YH, Hamadah K. Effect of ADP on PGE1 formation in blood platelets from patients with depression, mania and schizophrenia. Br J Psychiatry. 127:591–5.

59. Adibhatla RM, Hatcher JF, Dempsey RJ. Lipids and lipidomics in brain injury and diseases. AAPS J. 8(2):E314-321.

60. Adibhatla RM, Hatcher JF. Role of Lipids in Brain Injury and Diseases. Future Lipidol. 2(4):403–22.

61. Adibhatla RM, Hatcher JF. Altered lipid metabolism in brain injury and disorders. Subcell Biochem. 2008;49:241–68.

62. Akarsu S, Bolu A, Aydemir E, Zincir SB, Kurt YG, Zincir S, et al. The Relationship between the Number of Manic Episodes and Oxidative Stress Indicators in Bipolar Disorder. Psychiatry Investig. 15(5):514–9.

63. Akimoto T. Study on the mechanism to maintain calcium mobilization in platelets of patients with bipolar affective disorders–the relationship with the protein kinase C system. Hokkaido Igaku Zasshi - Hokkaido J Med Sci. 79(5):597–607.

64. Akyol O, Chowdhury I, Akyol HR, Tessier K, Vural H, Akyol S. Why are cardiovascular diseases more common among patients with severe mental illness? The potential involvement of electronegative low-density lipoprotein (LDL) L5. Med Hypotheses. 142:109821.

65. Alam R, Abdolmaleky HM, Zhou JR. Microbiome, inflammation, epigenetic alterations, and mental diseases. Am J Med Genet Part B Neuropsychiatr Genet Off Publ Int Soc Psychiatr Genet. 174(6):651–60.

66. Alarcon RD, Tolbert LC, Monti JA, Morere DA, Walter-Ryan WG, Kemp B, et al. One-carbon metabolism disturbances in affective disorders. A preliminary report. J Affect Disord. 9(3):297–301.

67. Ande S, Sawarkar H, Bakal R, Ch, ewar AV, Kshirsagar MD. Lipids and stress: A correlation in neurodegenerative disorders. Int J Pharm Sci Rev Res. 57(1):43–8.

68. Andreassen OA, Desikan RS, Wang Y, Thompson WK, Schork AJ, Zuber V, et al. Abundant genetic overlap between blood lipids and immune-mediated diseases indicates shared molecular genetic mechanisms. PloS One. 2015;10(4):e0123057.

69. Arnsten A, Manji H. Mania: a rational neurobiology. Future Neurol. 2008 Mar;3(2):125–31.

70. Astorg P. Omega-3 polyunsaturated fatty acids and mood disorders. OCL - Ol Corps Gras Lipides. 2007;14(3):202–7.

71. Atagun MI, Tunc S, Alisik M, Erel O. Phenotypic Variants of Paraoxonase Q192R in Bipolar Disorder,Depression and Schizophrenia. Turk Psikiyatri Derg. 2018;29(2):79–86.

72. Bai YM, Chen MH, Hsu JW, Huang KL, Tu PC, Chang WC, et al. A comparison study of metabolic profiles, immunity, and brain gray matter volumes between patients with bipolar disorder and depressive disorder. J Neuroinflammation. 17(1):42.

73. Baptista T, S, ia I, Fern, ez E, Balzan L, et al. Metabolic syndrome and related variables, insulin resistance, leptin levels, and PPAR-gamma2 and leptin gene polymorphisms in a pedigree of subjects with bipolar disorder. Rev Bras Psiquiatr. 2015;37(2):106–12.

74. Bartoli F, Di Brita C, Crocamo C, Clerici M, Carra G. Lipid profile and suicide attempt in bipolar disorder: A meta-analysis of published and unpublished data. Prog Neuropsychopharmacol Biol Psychiatry. 79:90–5.

75. Bavaresco DV, Uggioni MLR, Simon CS, Colonetti T, Ferraz SD, Cruz MVB, et al. Evaluation of the arachidonic acid pathway in bipolar disorder: a systematic review. Mol Biol Rep. 47(10):8209–17.

76. Beasley CL, Honer WG, Bergmann K, Falkai P, Lutjohann D, Bayer TA. Reductions in cholesterol and synaptic markers in association cortex in mood disorders. Bipolar Disord. 7(5):449–55.

77. Beasley CL, Honer WG, Ramos-Miguel A, Vila-Rodriguez F, Barr AM. Prefrontal fatty acid composition in schizophrenia and bipolar disorder: Association with reelin expression. Schizophr Res. 215:493–8.

78. Bellivier F, Laplanche JL, Schurhoff F, Feingold J, Feline A, Jouvent R, et al. Apolipoprotein E gene polymorphism in early and late onset bipolar patients. Neurosci Lett. 233(1):45–8.

79. Bennett CN, Horrobin DF. Gene targets related to phospholipid and fatty acid metabolism in schizophrenia and other psychiatric disorders: an update. Prostaglandins Leukot Essent Fatty Acids. 63(1):47–59.

80. Bly MJ, Taylor SF, Dalack G, Pop-Busui R, Burghardt KJ, Evans SJ, et al. Metabolic syndrome in bipolar disorder and schizophrenia: Dietary and lifestyle factors compared to the general population. Bipolar Disord. 16(3):277–88.

81. Bocchetta A, Chillotti C, Carboni G, Oi A, Ponti M, Del Zompo M. Association of personal and familial suicide risk with low serum cholesterol concentration in male lithium patients. Acta Psychiatr Scand. 104(1):37–41.

82. L, ucci Bonifacio K, Sabbatini Barbosa D, Gastaldello Moreira E, de Farias CC, Higachi L, et al. Indices of insulin resistance and glucotoxicity are not associated with bipolar disorder or major depressive disorder, but are differently associated with inflammatory, oxidative and nitrosative biomarkers. J Affect Disord. 222:185–94.

83. Bonifacio KL, Barbosa DS, Moreira EG, Coneglian CF, Vargas HO, Nunes SOV, et al. Increased nitro-oxidative stress toxicity as a major determinant of increased blood pressure in mood disorders. J Affect Disord. 278:226–38.

84. Bortolasci CC, Vargas HO, Souza-Nogueira A, Barbosa DS, Moreira EG, Nunes SO, et al. Lowered plasma paraoxonase (PON)1 activity is a trait marker of major depression and PON1 Q192R gene polymorphism-smoking interactions differentially predict the odds of major depression and bipolar disorder. J Affect Disord. 159:23–30.

85. Bortolasci CC, Vargas HO, Vargas Nunes SO, de Melo LG, de Castro MR, Moreira EG, et al. Factors influencing insulin resistance in relation to atherogenicity in mood disorders, the metabolic syndrome and tobacco use disorder. J Affect Disord. 179:148–55.

86. Br, rup E, R, rup A. A controlled investigation of plasma lipids in manic-depressives. Br J Psychiatry. 113(502):987–92.

87. Bristot G, De Bastiani MA, Pfaffenseller B, Kapczinski F, Kauer-Sant'Anna M. Gene Regulatory Network of Dorsolateral Prefrontal Cortex: a Master Regulator Analysis of Major Psychiatric Disorders. Mol Neurobiol. 57(3):1305–16.

88. Brown AS, Mallinger AG, Renbaum LC. Elevated platelet membrane phosphatidylinositol-4,5-bisphosphate in bipolar mania. Am J Psychiatry. 150(8):1252–4.

89. Brown NC, Andreazza AC, Young LT. An updated meta-analysis of oxidative stress markers in bipolar disorder. Psychiatry Res. 218(1):61–8.

90. Burghardt KJ, Gardner KN, Johnson JW, Ellingrod VL. Fatty Acid desaturase gene polymorphisms and metabolic measures in schizophrenia and bipolar patients taking antipsychotics. Cardiovasc Psychiatry Neurol. 2013;2013:596945.

91. Campbell I, Campbell H. A pyruvate dehydrogenase complex disorder hypothesis for bipolar disorder. Med Hypotheses. 130:109263.

92. Ceylan D, Tuna G, Kirkali G, Tunca Z, Can G, Arat HE, et al. Oxidatively-induced DNA damage and base excision repair in euthymic patients with bipolar disorder. DNA Repair. 65:64–72.

93. Ceylan D, Tufekci KU, Keskinoglu P, Genc S, Ozerdem A. Circulating exosomal microRNAs in bipolar disorder. J Affect Disord. 262:99–107.

94. Chang YW, Assari S, Prossin AR, Stertz L, McInnis MG, Evans SJ. Bipolar disorder moderates associations between linoleic acid and markers of inflammation. J Psychiatr Res. 85:29–36.

95. Chiurchiu V, Maccarrone M. Bioactive lipids as modulators of immunity, inflammation and emotions. Curr Opin Pharmacol. 29:54–62.

96. Chowdhury MI, Hasan M, Islam MS, Sarwar MS, Amin MN, Uddin SMN, et al. Elevated serum MDA and depleted non-enzymatic antioxidants, macro-minerals and trace elements are associated with bipolar disorder. J Trace Elem Med Biol. 39:162–8.

97. Clark SR, Baune BT, Schubert KO, Lavoie S, Smesny S, Rice SM, et al. Prediction of transition from ultra-high risk to first-episode psychosis using a probabilistic model combining history, clinical assessment and fatty-acid biomarkers. Transl Psychiatry Psychiatry. 6(9):e897.

98. Clayton EH, Hanstock TL, Hirneth SJ, Kable CJ, Garg ML, Hazell PL. Long-chain omega-3 polyunsaturated fatty acids in the blood of children and adolescents with juvenile bipolar disorder. Lipids. 43(11):1031–8.

99. Cocchi M, Gabrielli F, Pessa E, Pregnolato M, Tonello L, Zizzi P. Major depression and bipolar disorder: The concept of symmetry breaking. NeuroQuantology. 2012;10(4):676–87.

100. Cocchi M, Tonello L, Gabrielli F. Mood psychopathologies: An integrated complexity-based interpretation. Psychology. 5(3):192–203.

101. Cocchi M, Minuto C, Tonello L, Gabrielli F, Bernroider G, Tuszynski JA, et al. Linoleic acid: Is this the key that unlocks the quantum brain? Insights linking broken symmetries in molecular biology, mood disorders and personalistic emergentism. BMC Neurosci [Internet]. 2017;18(1). Available from: http://www.biomedcentral.com/bmcneurosci/

102. Coryell W, Schlesser M. Combined biological tests for suicide prediction. Psychiatry Res. 150(2):187–91.

103. Cudney LE, Sassi RB, Behr GA, Streiner DL, Minuzzi L, Moreira JC, et al. Alterations in circadian rhythms are associated with increased lipid peroxidation in females with bipolar disorder. Int J Neuropsychopharmacol. 17(5):715–22.

104. Cuellar-Barboza AB, Cabello-Arreola A, Winham SJ, Colby C, Romo-Nava F, Nunez NA, et al. Body mass index and blood pressure in bipolar patients: Target cardiometabolic markers for clinical practice. J Affect Disord. 282:637–43.

105. Cuturic M, Abramson RK, Breen RJ, Edwards AC, Levy EE. Comparison of serum carnitine levels and clinical correlates between outpatients and acutely hospitalised individuals with bipolar disorder and schizophrenia: A cross-sectional study. World J Biol Psychiatry. 17(6):475–9.

106. Cyrino LAR, Delwing-de Lima D, Ullmann OM, Maia TP. Concepts of Neuroinflammation and Their Relationship With Impaired Mitochondrial Functions in Bipolar Disorder. Front Behav Neurosci. 2021;15:609487.

107. Dalkner N, Platzer M, Bengesser SA, Birner A, Fellendorf FT, Queissner R, et al. The role of tryptophan metabolism and food craving in the relationship between obesity and bipolar disorder. Clin Nutr. 37(5):1744–51.

108. Davison KM, Kaplan BJ. Food intake and blood cholesterol levels of community-based adults with mood disorders. BMC Psychiatry. 12:10.

109. De Berardis D, Conti CMV, Serroni N, Moschetta FS, Carano A, Salerno RM, et al. The role of cholesterol levels in mood disorders and suicide. J Biol Regul Homeost Agents. 23(3):133–40.

110. Dean B, Digney A, Thomas E, Scarr E. 09-03 The role of apolipoprotein E in the pathology of schizophrenia and bipolar disorder. Acta Neuropsychiatr. 18(6):334.

111. Dean B, Digney A, Sundram S, Thomas E, Scarr E. Plasma apolipoprotein E is decreased in schizophrenia spectrum and bipolar disorder. Psychiatry Res. 158(1):75–8.

112. Dieset I, Morch RH, Hope S, Hoseth EZ, Reponen EJ, Gran JM, et al. An association between YKL-40 and type 2 diabetes in psychotic disorders. Acta Psychiatr Scand. 139(1):37–45.

113. Dolapoglu N, Yurekli BPS, Eker MC, Elbi H. Relationship Between Serum Agouti-Related Peptide Levels and Metabolic Syndrome in Euthymic Bipolar Patients. Noropsikiyatri Arsivi. 58(1):16–20.

114. Dumas ME, Davidovic L. Metabolic phenotyping and systems biology approaches to understanding neurological disorders. F1000Prime Rep [Internet]. 5. Available from: http://f1000.com/prime/reports/b/5/18/pdf

115. Elvsashagen T, Zuzarte P, Westlye LT, Boen E, Josefsen D, Boye B, et al. Dentate gyrus-cornu ammonis (CA) 4 volume is decreased and associated with depressive episodes and lipid peroxidation in bipolar II disorder: Longitudinal and cross-sectional analyses. Bipolar Disord. 18(8):657–68.

116. Emanuele E, Carlin MV, D'Angelo A, Peros E, Barale F, Geroldi D, et al. Elevated plasma levels of lipoprotein(a) in psychiatric patients: a possible contribution to increased vascular risk. Eur Psychiatry J Assoc Eur Psychiatr. 21(2):129–33.

117. Erkiran M, Evren C, Guzelhan Y, Erkiran G. The aggression in manic inpatients and the relationship with blood cholesterol levels: A controlled study. Psikiyatri Psikol Psikofarmakol Derg. 2002;10(3):221–8.

118. Ersan S, Kurt A. Evaluation of glucagon-like peptide-1, adropin, and desnutrin levels and related factors in patients with bipolar disorder. Anadolu Psikiyatri Derg. 2021;22(1):1–6.

119. Ertekin H, Sahin B, Caliskan AM, Inanli I, Ertekin YH. Metabolic syndrome and vaspin in patients with bipolar disorder. Kaohsiung J Med Sci. 34(9):522–8.

120. Ezzaher A, Haj Mouhamed D, Mechri A, Neffati F, Douki W, Gaha L, et al. Obesity and dyslipidemia in Tunisian bipolar subjects. Ann Biol Clin (Paris). 68(3):277–84.

121. Ezzaher A, Haj Mouhamed D, Mechri A, Neffati F, Douki W, Gaha L, et al. Thyroid function and lipid profile in bipolar I patients. Asian J Psychiatry. 4(2):139–43.

122. Fagiolini A, Chengappa KN, Soreca I, Chang J. Bipolar disorder and the metabolic syndrome: causal factors, psychiatric outcomes and economic burden. CNS Drugs. 2008;22(8):655–69.

123. Farstad M. Determination qf fatty acids in cerebrospinal fluid. v. the fatty acid content in the total lipids of cerebrospinal fluid in psychiatric patients. Scand J Clin Lab Invest. 1966;18(3):343–6.

124. Fe'li SN, Ardekani SMY, Dehghani A. Relationship between serum homocysteine and metabolic syndrome among patients with schizophrenia and bipolar disorder: A cross sectional study. Iran J Psychiatry. 2020;15(4):266–73.

125. Fern, es BS, Karmakar C, Tamouza R, Tran T, Yearwood J, et al. Precision psychiatry with immunological and cognitive biomarkers: a multi-domain prediction for the diagnosis of bipolar disorder or schizophrenia using machine learning. Transl Psychiatry [Internet]. 2020;10(1). Available from: http://www.nature.com/tp/index.html

126. Fiedorowicz JG, Coryell WH. Cholesterol and suicide attempts: a prospective study of depressed inpatients. Psychiatry Res. 152(1):11–20.

127. Fiedorowicz JG, Palagummi NM, Behrendtsen O, Coryell WH. Cholesterol and affective morbidity. Psychiatry Res. 175(1):78–81.

128. Fleet-Michaliszyn SB, Soreca I, Otto AD, Jakicic JM, Fagiolini A, Kupfer DJ, et al. A prospective observational study of obesity, body composition, and insulin resistance in 18 women with bipolar disorder and 17 matched control subjects. J Clin Psychiatry. 69(12):1892–900.

129. Foguet-Boreu Q, Guardia Sancho A, Santos Lopez JM, Roura Poch P, Palmarola Ginesta J, Puig-Ribera AM, et al. Association between cognitive impairment and cardiovascular burden in patients with severe mental disorder. Cognit Neuropsychiatry. 25(1):1–13.

130. Freeman MP, Rapaport MH. Omega-3 fatty acids and depression: from cellular mechanisms to clinical care. J Clin Psychiatry. 72(2):258–9.

131. Frey BN, Stanley JA, Nicoletti MA, Hatch JP, Soares JC. Corrected values of brain metabolites for the article: "Abnormal cellular energy and phospholipid metabolism in the

left dorsolateral prefrontal cortex of medication-free individuals with bipolar disorder: An in vivo 1H MRS study.” Bipolar Disord. 10(7):849.

132. Frey BN, Stanley JA, Nery FG, Serap Monkul E, Nicoletti MA, Chen HH, et al. Abnormal cellular energy and phospholipid metabolism in the left dorsolateral prefrontal cortex of medication-free individuals with bipolar disorder: An in vivo 1 H MRS study. Bipolar Disord Suppl. 2007;9(1):119–27.

133. Fries GR, Quevedo J, Zeni CP, Kazimi IF, Zunta-Soares G, Spiker DE, et al. Integrated transcriptome and methylome analysis in youth at high risk for bipolar disorder: A preliminary analysis. Transl Psychiatry [Internet]. 2017;7(3). Available from: https://www.scopus.com/inward/record.uri?eid=2-s2.0-85037588602&doi=10.1038%2ftp.2017.32&partnerID=40&md5=5b92b8a600aa32eb548e52a61488ad4d

134. Geoffroy PA, Godin O, Mahee D, Henry C, Aubin V, Azorin JM, et al. Seasonal pattern in bipolar disorders and cardio-vascular risk factors: A study from the FACE-BD cohort. Chronobiol Int. 2017;34(7):845–54.

135. Ghosh S, Dyer RA, Beasley CL. Evidence for altered cell membrane lipid composition in postmortem prefrontal white matter in bipolar disorder and schizophrenia. J Psychiatr Res. 95:135–42.

136. Glen AI, Cooper SJ, Rybakowski J, Vaddadi K, Brayshaw N, Horrobin DF. Membrane fatty acids, niacin flushing and clinical parameters. Prostaglandins Leukot Essent Fatty Acids. 55(1):9–15.

137. Godin O, Henry C, Leboyer M, Azorin JM, Aubin V, Bellivier F, et al. Sleep quality, chronotype and metabolic syndrome components in bipolar disorders during the remission period: Results from the FACE-BD cohort. Chronobiol Int. 2017;34(8):1114–24.

138. Golkowski M, Perera GK, Vidadala VN, Ojo KK, Van Voorhis WC, Maly DJ, et al. Kinome chemoproteomics characterization of pyrrolo 3,4-c pyrazoles as potent and selective inhibitors of glycogen synthase kinase 3. Mol Omics. 14(1):26–36.

139. Gubert C, Stertz L, Pfaffenseller B, Panizzutti BS, Rezin GT, Massuda R, et al. Mitochondrial activity and oxidative stress markers in peripheral blood mononuclear cells of patients with bipolar disorder, schizophrenia, and healthy subjects. J Psychiatr Res. 47(10):1396–402.

140. Hamazaki K, Choi KH, Kim HY. Phospholipid profile in the postmortem hippocampus of patients with schizophrenia and bipolar disorder: no changes in docosahexaenoic acid species. J Psychiatr Res. 44(11):688–93.

141. Hamazaki K, Hamazaki T, Inadera H. Fatty acid composition in the postmortem amygdala of patients with schizophrenia, bipolar disorder, and major depressive disorder. J Psychiatr Res. 46(8):1024–8.

142. Hamazaki K, Hamazaki T, Inadera H. Polyunsaturated fatty acids and their related enzymes in postmortem brain tissues of patients with psychiatric disorders. Curr Psychopharmacol. 2013;2(1):66–72.

143. Hamazaki K, Hamazaki T, Inadera H. Abnormalities in the fatty acid composition of the postmortem entorhinal cortex of patients with schizophrenia, bipolar disorder, and major depressive disorder. Psychiatry Res. 210(1):346–50.

144. Hamazaki K, Maekawa M, Toyota T, Dean B, Hamazaki T, Yoshikawa T. Fatty acid composition of the postmortem prefrontal cortex of patients with schizophrenia, bipolar disorder, and major depressive disorder. Psychiatry Res. 227(2):353–9.

145. Hamazaki K, Maekawa M, Toyota T, Dean B, Hamazaki T, Yoshikawa T. Fatty acid composition of the postmortem corpus callosum of patients with schizophrenia, bipolar disorder, or major depressive disorder. Eur Psychiatry J Assoc Eur Psychiatr. 39:51–6.

146. Hammond GR, Schiavo G. Polyphosphoinositol lipids: under-PPInning synaptic function in health and disease. Dev Neurobiol. 67(9):1232–47.

147. Hashimoto K. Metabolomics of Major Depressive Disorder and Bipolar Disorder: Overview and Future Perspective. Adv Clin Chem. 2018;84:81–99.

148. Hatch J, Andreazza A, Olowoyeye O, Rezin GT, Moody A, Goldstein BI. Cardiovascular and psychiatric characteristics associated with oxidative stress markers among adolescents with bipolar disorder. J Psychosom Res. 79(3):222–7.

149. Horrobin DF. The possible roles of prostaglandin E1 and of essential fatty acids in mania, depression and alcoholism. Prog Lipid Res. 1981;20:539–41.

150. Horrobin DF, Bennett CN. Depression and bipolar disorder: relationships to impaired fatty acid and phospholipid metabolism and to diabetes, cardiovascular disease, immunological abnormalities, cancer, ageing and osteoporosis. Possible candidate genes. Prostaglandins Leukot Essent Fatty Acids. 60(4):217–34.

151. Horrobin DF, Bennett CN. New gene targets related to schizophrenia and other psychiatric disorders: enzymes, binding proteins and transport proteins involved in phospholipid and fatty acid metabolism. Prostaglandins Leukot Essent Fatty Acids. 60(3):141–67.

152. Howells FM, Ives-Deliperi VL, Horn NR, Stein DJ. Increased thalamic phospholipid concentration evident in bipolar I disorder. Prog Neuropsychopharmacol Biol Psychiatry. 41:1–5.

153. Hu Q, Wang C, Liu F, He J, Wang F, Wang W, et al. High serum levels of FGF21 are decreased in bipolar mania patients during psychotropic medication treatment and are associated with increased metabolism disturbance. Psychiatry Res. 272:643–8.

154. Hullin RP, Court G. Fasting blood lipid concentrations>> in manic depressive psychosis. BritJPsychiat. 1970;117(538):275–85.

155. Hylén U, McGlinchey A, Orešič M, Bejerot S, Humble MB, Särndahl E, et al. Potential Transdiagnostic Lipid Mediators of Inflammatory Activity in Individuals With Serious Mental Illness. Front Psychiatry. 2021;12:778325.

156. Igarashi M, Ma K, Gao F, Kim HW, Greenstein D, Rapoport SI, et al. Brain lipid concentrations in bipolar disorder. J Psychiatr Res. 2010 Feb 1;44(3):177–82.

157. Ikenaga EH, Talib LL, Ferreira AS, Machado-Vieira R, Forlenza OV, Gattaz WF. Reduced activities of phospholipases A2 in platelets of drug-naive bipolar disorder patients. Bipolar Disord. 17(1):97–101.

158. Jakovljevic M, Reiner Z, Milicic D. Mental disorders, treatment response, mortality and serum cholesterol: a new holistic look at old data. Psychiatr Danub. 19(4):270–81.

159. Joshi YB, Pratico D. Lipid peroxidation in psychiatric illness: overview of clinical evidence. Oxid Med Cell Longev. 2014;2014:828702.

160. Kalelioglu T, Unalan P, Kok B, Sozen S, Yuksel O, Akkus M, et al. Atherogenic index of plasma as a cardiovascular risk marker in manic, depressive, and euthymic stages of bipolar disorder. Turk Kardiyol Dernegi Arsivi. 46(1):32–8.

161. Kapczinski F, Frey BN, Andreazza AC, Kauer-Sant'Anna M, Cunha AB, Post RM. Increased oxidative stress as a mechanism for decreased BDNF levels in acute manic episodes. Rev Bras Psiquiatr. 30(3):243–5.

162. Kavoor AR, Mitra S, Ram D. Gender difference in lipid profile in bipolar disorder: Indian scenario. Asian J Psychiatry. 18:104–5.

163. Kenna HA, Jiang B, Rasgon NL. Reproductive and metabolic abnormalities associated with bipolar disorder and its treatment. Harv Rev Psychiatry. 2009;17(2):138–46.

164. Kenneson A, Funderburk JS. Patatin-like phospholipase domain-containing protein 3 (PNPLA3): A potential role in the association between liver disease and bipolar disorder. J Affect Disord. 2017;209:93–6.

165. Kesebir S, Erdinc B, Tarhan N. Predictors of metabolic syndrome in first manic episode. Asian J Psychiatry. 25:179–83.

166. Kilic EK, Cinar RK, Gorgulu Y, Sonmez MB. Increased mitochondrial and cytosolic antioxidant enzymes in manic episodes. Eur J Psychiatry. 34(4):181–8.

167. Kim B, Kim S, McIntyre RS, Hui JP, Seong YK, Yeon HJ. Correlates of metabolic abnormalities in bipolar I disorder at initiation of acute phase treatment. Psychiatry Investig. 6(2):78–84.

168. Kim EY, Kim SH, Lee HJ, Kim B, Kim YS, Ahn YM. Sex-specific association between the albumin D-element binding protein gene and metabolic syndrome in patients with bipolar disorder and schizophrenia. Psychiatry Res. 240:47–52.

169. Knochel C, Kniep J, Cooper JD, Stablein M, Wenzler S, Sarlon J, et al. Altered apolipoprotein C expression in association with cognition impairments and hippocampus volume in schizophrenia and bipolar disorder. Eur Arch Psychiatry Clin Neurosci. 267(3):199–212.

170. Knowles EE, Meikle PJ, Huynh K, Goring HH, Olvera RL, Mathias SR, et al. Serum phosphatidylinositol as a biomarker for bipolar disorder liability. Bipolar Disord. 19(2):107–15.

171. Kovaleva ES, Orlov ON, Piatnitskii AN, Beliaev BS, Erin AN. Dynamics of the lipid peroxidation process in the body of patients with manic-depressive psychosis. Zhurnal Nevropatol Psikhiatrii Im - - Korsakova. 1988;88(4):69–71.

172. Kulak-Bejda A, Bejda G, Lech M, Waszkiewicz N. Are Lipids Possible Markers of Suicide Behaviors? J Clin Med. 10(2):18.

173. Kuloglu M, Ustundag B, Atmaca M, Canatan H, Tezcan AE, Cinkilinc N. Lipid peroxidation and antioxidant enzyme levels in patients with schizophrenia and bipolar disorder. Cell Biochem Funct. 2002 Jun;20(2):171–5.

174. Kurt E, Altinbas K, Alatas G, Yesilbas D, Bilgic V, Ozver I. Are bipolar I patients different from schizophrenic patients in terms of diabetes and dyslipidemia comorbidity? Klin Psikiyatri Derg J Clin Psychiatry. 2009;12(2):72–8.

175. Kusumi I, Arai Y, Okubo R, Honda M, Matsuda Y, Matsuda Y, et al. Predictive factors for hyperglycaemic progression in patients with schizophrenia or bipolar disorder. BJPsych Open. 4(6):454–60.

176. Lang F, Ma K, Leibrock CB. 1,25$(OH)_2D_3$ in Brain Function and Neuropsychiatric Disease. NeuroSignals. 2019;27(1):40–9.

177. Lange KW. Omega-3 fatty acids and mental health. Glob Health J. 2020 Mar 1;4(1):18–30.

178. Le-Niculescu H, Case NJ, Hulvershorn L, Patel SD, Bowker D, Gupta J, et al. Convergent functional genomic studies of omega-3 fatty acids in stress reactivity, bipolar disorder and alcoholism. Transl Psychiatry Psychiatry. 1:e4.

179. Lee H, Rhee SJ, Kim J, Lee Y, Kim H, Lee J, et al. Predictive protein markers for depression severity in mood disorders: A preliminary trans-diagnostic approach study. J Psychiatr Res. 2021 Oct;142:63–72.

180. Lengvenyte A, Aouizerate B, Aubin V, Loftus J, Marlinge E, Belzeaux R, et al. Violent suicide attempt history in elderly patients with bipolar disorder: The role of sex, abdominal obesity, and verbal memory: Results from the FACE-BD cohort (FondaMental Advanced center of Expertise for Bipolar Disorders). J Affect Disord. 2022 Jan 1;296:265–76.

181. Levey DF, Niculescu EM, Le-Niculescu H, Dainton HL, Phalen PL, Ladd TB, et al. Towards understanding and predicting suicidality in women: biomarkers and clinical risk assessment. Mol Psychiatry. 21(6):768–85.

182. Li Z, Lai J, Zhang P, Ding J, Jiang J, Liu C, et al. Multi-omics analyses of serum metabolome, gut microbiome and brain function reveal dysregulated microbiota-gut-brain axis in bipolar depression. Mol Psychiatry. 2022 Apr 20;

183. Liao PJ, Chen CH, Chan HY, Tan HK, Hsu KH. Serum lipid profile could predict the inception and impacts of violent behaviors among acute psychiatric inpatients. Chang Gung Med J. 35(5):382–91.

184. Liu JJ, Green P, John Mann J, Rapoport SI, Sublette ME. Pathways of polyunsaturated fatty acid utilization: implications for brain function in neuropsychiatric health and disease. Brain Res. 1597:220–46.

185. Lopuszanska U, Sidor K, Makara-Studzinska M. Psychosocial determinants of metabolic disorders in individuals with psychiatric disorders. Psychiatr Psychol Klin. 17(1):17–22.

186. Lunding SH, Simonsen C, Aas M, Rodev, L., Werner MCF, et al. Childhood trauma and cardiometabolic risk in severe mental disorders: The mediating role of cognitive control. Eur Psychiatry J Assoc Eur Psychiatr. 64(1):e24.

187. Maes M, L, ucci Bonifacio K, Morelli NR, Vargas HO, Barbosa DS, et al. Major Differences in Neurooxidative and Neuronitrosative Stress Pathways Between Major Depressive Disorder and Types I and II Bipolar Disorder. Mol Neurobiol. 56(1):141–56.

188. Maida ME, Hurley SD, Daeschner JA, Moore AH, O'Banion MK. Cytosolic prostaglandin E2 synthase (cPGES) expression is decreased in discrete cortical regions in psychiatric disease. Brain Res. 1103(1):164–72.

189. Mallick R, Basak S, Duttaroy AK. Docosahexaenoic acid,22:6n-3: Its roles in the structure and function of the brain. Int J Dev Neurosci. 79:21–31.

190. Mansur RB, Rizzo LB, Santos CM, Asevedo E, Cunha GR, Noto MN, et al. Adipokines, metabolic dysfunction and illness course in bipolar disorder. J Psychiatr Res. 74:63–9.

191. Margari F, Lozupone M, Pisani R, Pastore A, Todarello O, Zagaria G, et al. Metabolic syndrome: differences between psychiatric and internal medicine patients. Int J Psychiatry Med. 2013;45(3):203–26.

192. McNamara RK, Ostr, er M, Abplanalp W, Richt, N. M., et al. Modulation of phosphoinositide-protein kinase C signal transduction by omega-3 fatty acids: implications for the pathophysiology and treatment of recurrent neuropsychiatric illness. Prostaglandins Leukot Essent Fatty Acids. 75(4):237–57.

193. McNamara RK, J, acek R, Rider T, Tso P, Dwivedi Y, et al. Selective deficits in erythrocyte docosahexaenoic acid composition in adult patients with bipolar disorder and major depressive disorder. J Affect Disord. 126(1):303–11.

194. McNamara RK, Lotrich FE. Elevated immune-inflammatory signaling in mood disorders: a new therapeutic target? Expert Rev Neurother. 12(9):1143–61.

195. McNamara RK. Long-chain omega-3 fatty acid deficiency in mood disorders: rationale for treatment and prevention. Curr Drug Discov Technol. 10(3):233–44.

196. McNamara RK, Rider T, J, acek R, Tso P. Abnormal fatty acid pattern in the superior temporal gyrus distinguishes bipolar disorder from major depression and schizophrenia and resembles multiple sclerosis. Psychiatry Res. 215(3):560–7.

197. McNamara RK. Mitigation of Inflammation-Induced Mood Dysregulation by Long-Chain Omega-3 Fatty Acids. J Am Coll Nutr. 2015;34:48–55.

198. Meira-Lima IV, Vallada H. Genes related to phospholipid metabolism as risk factors related to bipolar affective disorder. Rev Bras Psiquiatr. 25(1):51–5.

199. Messamore E, Almeida DM, J, acek RJ, McNamara RK. Polyunsaturated fatty acids and recurrent mood disorders: Phenomenology, mechanisms, and clinical application. Prog Lipid Res. 66:1–13.

200. Messamore E, McNamara RK. Detection and treatment of omega-3 fatty acid deficiency in psychiatric practice: Rationale and implementation. Lipids Health Dis. 15:25.

201. Mico JA, Rojas-Corrales MO, Gibert-Rahola J, Parellada M, Moreno D, Fraguas D, et al. Reduced antioxidant defense in early onset first-episode psychosis: A case-control study. BMC Psychiatry [Internet]. 11. Available from: http://www.biomedcentral.com/1471-244X/11/26

202. Moolamalla STR, Vinod PK. Genome-scale metabolic modelling predicts biomarkers and therapeutic targets for neuropsychiatric disorders. Comput Biol Med. 125:103994.

203. Morris G, Puri BK, Bortolasci CC, Carvalho A, Berk M, Walder K, et al. The role of high-density lipoprotein cholesterol, apolipoprotein A and paraoxonase-1 in the pathophysiology of neuroprogressive disorders. Neurosci Biobehav Rev. 125:244–63.

204. Morris G, Puri BK, Walker AJ, Maes M, Carvalho AF, Bortolasci CC, et al. Shared pathways for neuroprogression and somatoprogression in neuropsychiatric disorders. Neurosci Biobehav Rev. 107:862–82.

205. Muskiet FA, Kemperman RF. Folate and long-chain polyunsaturated fatty acids in psychiatric disease. J Nutr Biochem. 17(11):717–27.

206. Nasca C, Barnhill O, DeAngelis P, Watson K, Lin J, Beasley J, et al. Multidimensional predictors of antidepressant responses: Integrating mitochondrial, genetic, metabolic and environmental factors with clinical outcomes. Neurobiol Stress. 2021 Nov;15:100407.

207. Newton DF, Naiberg MR, Andreazza AC, Scola G, Dickstein DP, Goldstein BI. Association of Lipid Peroxidation and Brain-Derived Neurotrophic Factor with Executive Function in Adolescent Bipolar Disorder. Psychopharmacology (Berl). 234(4):647–56.

208. Omileke F, Ishiwata S, Matsuo J, Yoshida F, Hidese S, Hattori K, et al. Possible associations between plasma fibroblast growth factor 21 levels and cognition in bipolar disorder. Neuropsychopharmacol Rep. 40(2):175–81.

209. Ong WY, Farooqui T, Kokotos G, Farooqui AA. Synthetic and natural inhibitors of phospholipases A2: their importance for understanding and treatment of neurological disorders. Acs Chem Neurosci. 6(6):814–31.

210. Ozcan ME, Gulec M, Ozerol E, Polat R, Akyol O. Antioxidant enzyme activities and oxidative stress in affective disorders. Int Clin Psychopharmacol. 19(2):89–95.

211. Patrick RP, Ames BN. Vitamin D and the omega-3 fatty acids control serotonin synthesis and action, part 2: relevance for ADHD, bipolar disorder, schizophrenia, and impulsive behavior. FASEB J. 29(6):2207–22.

212. Pedrini M, Massuda R, Fries GR, de Bittencourt Pasquali MA, Schnorr CE, Moreira JC, et al. Similarities in serum oxidative stress markers and inflammatory cytokines in patients with overt schizophrenia at early and late stages of chronicity. J Psychiatr Res. 46(6):819–24.

213. Persons JE, Coryell WH, Fiedorowicz JG. Cholesterol fractions, symptom burden, and suicide attempts in mood disorders. Psychiatry Res. 200(2):1088–9.

214. Plana T, Gracia R, Mendez I, Pintor L, Lazaro L, Castro-Fornieles J. Total serum cholesterol levels and suicide attempts in child and adolescent psychiatric inpatients. Eur Child Adolesc Psychiatry. 19(7):615–9.

215. Quintero M, Stanisic D, Cruz G, Pontes JGM, Costa TBBC, Tasic L. Metabolomic Biomarkers in Mental Disorders: Bipolar Disorder and Schizophrenia. Adv Exp Med Biol. 2019;1118:271–93.

216. Reponen EJ, Dieset I, Tesli M, Morch RH, Aas M, Vedal TSJ, et al. Atherogenic Lipid Ratios Related to Myeloperoxidase and C-Reactive Protein Levels in Psychotic Disorders. Front Psychiatry Front Res Found. 2020;11:672.

217. Roedding AS, Li PP, Warsh JJ. Characterization of the transient receptor potential channels mediating lysophosphatidic acid-stimulated calcium mobilization in B lymphoblasts. Life Sci. 80(2):89–97.

218. Ross BM, Hughes B, Kish SJ, Warsh JJ. Serum calcium-independent phospholipase A2 activity in bipolar affective disorder. Bipolar Disord. 8(3):265–70.

219. Rosso G, Cattaneo A, Zanardini R, Gennarelli M, Maina G, Bocchio-Chiavetto L. Glucose metabolism alterations in patients with bipolar disorder. J Affect Disord. 184:293–8.

220. Russell A, Ciufolini S, Gardner-Sood P, Bonaccorso S, Gaughran F, Dazzan P, et al. Inflammation and metabolic changes in first episode psychosis: preliminary results from a longitudinal study. Brain Behav Immun. 49:25–9.

221. Rutkofsky IH, Khan AS, Sahito S, Kumar V. The Psychoneuroimmunological Role of Omega-3 Polyunsaturated Fatty Acids in Major Depressive Disorder and Bipolar Disorder. Adv Mind Body Med. 31(3):8–16.

222. Rybakowski J, Ainiyet J, Szajnerman Z, Zakrzewska M. The study of the relationship between cholesterol and lipid concentration and suicidal behavior in patients with schizophrenia affective illness. Psychiatr Pol. 30(5):699–712.

223. Sagud M, Mihaljevic-Peles A, Pivac N, Jakovljevic M, Muck-Seler D. “Platelet serotonin and serum lipids in psychotic mania”: Corrigendum. J Affect Disord. 101(1):283.

224. Saugstad LF. Manic depressive psychosis and schizophrenia are neurological disorders at the extremes of CNS maturation and nutritional disorders associated with a deficit in marine fat. Med Hypotheses. 57(6):679–92.

225. Saugstad LF. Human nature is unique in the mismatch between the usual diet and the need for “food for the brain” (marine fat, DHA). Adding marine fat is beneficial in

schizophrenia and manic-depressive psychosis. This underlines brain dysfunction in these neurological disorders is associated with deficient intake of marine fat(DHA). Nutr Health. 2002;16(1):41–4.

226. Saugstad LF. Marine fat and brain function in manic-depressive psychosis and schizophrenia: circumstantial evidence for a 2nd aquatic period. Nutr Health. 2002;16(1):11–2.

227. Saunders EFH, Ramsden CE, Sherazy MS, Gelenberg AJ, Davis JM, Rapoport SI. Omega-3 and Omega-6 Polyunsaturated Fatty Acids in Bipolar Disorder: A Review of Biomarker and Treatment Studies. J Clin Psychiatry. 2016 Oct;77(10):e1301–8.

228. Schmechel DE. Art, alpha-1-antitrypsin polymorphisms and intense creative energy: blessing or curse? Neurotoxicology. 28(5):899–914.

229. Schneider M, Levant B, Reichel M, Gulbins E, Kornhuber J, Muller CP. Lipids in psychiatric disorders and preventive medicine. Neurosci Biobehav Rev. 76:336–62.

230. Schwarz E, Prabakaran S, Whitfield P, Major H, Leweke FM, Koethe D, et al. High throughput lipidomic profiling of schizophrenia and bipolar disorder brain tissue reveals alterations of free fatty acids, phosphatidylcholines, and ceramides. J Proteome Res. 2008 Oct;7(10):4266–77.

231. Scola G, Andreazza AC. Current State of Biomarkers in Bipolar Disorder. Curr Psychiatry Rep [Internet]. 2014;16(12). Available from: http://www.springerlink.com/content/1523-3812/

232. Scola G, McNamara RK, Croarkin PE, Leffler JM, Cullen KR, Geske JR, et al. Lipid peroxidation biomarkers in adolescents with or at high-risk for bipolar disorder. J Affect Disord. 192:176–83.

233. Setareh J, Hadinezhad P, Moosazadeh M, Masoudzadeh A, Mousavi SM, Ahmadi O. Changes in the indicators of metabolic syndrome in hospitalized psychiatric patients. J Mazandaran Univ Med Sci. 28(165):109–18.

234. Sethi S, Pedrini M, Rizzo LB, Zeni-Graiff M, Mas CD, Cassinelli AC, et al. ^{1}H-NMR, ^{1}H-NMR T<inf>2</inf>-edited, and 2D-NMR in bipolar disorder metabolic profiling. Int J Bipolar Disord [Internet]. 2017;5(1). Available from: http://www.journalbipolardisorders.com/

235. Shah R, Subhan F, Sultan SM, Haq M, Ahmad H, Khan QR, et al. Metabolic dysregulation in early onset psychiatric disorder before and after exposure to antipsychotic drugs. Braz J Pharm Sci [Internet]. 2019;55. Available from: http://www.scielo.br/pdf/bjps/v55/1984-8250-bjps-55-e17825.pdf

236. Shaheen O, Abdel-Rahman Y, Hanafi A, Erfan M. Some observations on lipid metabolism in mania patients. J Egypt Med Assoc. 1971;54(7):393–400.

237. Shamim A, Mahmood T, Ahsan F, Kumar A, Bagga P. Lipids: An insight into the neurodegenerative disorders. Clin Nutr Exp. 20:1–19.

238. Shapiro LR, Kennedy KG, Dimick MK, Goldstein BI. Elevated atherogenic lipid profile in youth with bipolar disorder during euthymia and hypomanic/mixed but not depressive states. J Psychosom Res. 2022 May;156:110763.

239. Smythies JR, Alarcon RD, Morere D, Monti JA, Steele M, Tolbert LC, et al. Abnormalities of one-carbon metabolism in psychiatric disorders: study of methionine adenosyltransferase kinetics and lipid composition of erythrocyte membranes. Biol Psychiatry. 21(14):1391–8.

240. So HC, Chau KL, Ao FK, Mo CH, Sham PC. Exploring shared genetic bases and causal relationships of schizophrenia and bipolar disorder with 28 cardiovascular and metabolic traits. Psychol Med. 49(8):1286–98.

241. Sobczak S, Honig A, Christophe A, Maes M, Helsdingen RW, De Vriese SA, et al. Lower high-density lipoprotein cholesterol and increased omega-6 polyunsaturated fatty acids in first-degree relatives of bipolar patients. Psychol Med. 34(1):103–12.

242. Sokol DK, O'Brien RS, Wagenknecht DR, Rao T, McIntyre JA. Antiphospholipid antibodies in blood and cerebrospinal fluids of patients with psychosis. J Neuroimmunol. 190(1):151–6.

243. Sonnik GT, Zazykina DS. Changes in various indices of the blood-clotting system and lipid levels in patients with depression. Zhurnal Nevropatol Psikhiatrii Im - - Korsakova. 1987;87(4):571–3.

244. Soreca I, Wallace ML, Frank E, Hasler BP, Levenson JC, Kupfer DJ. Sleep duration is associated with dyslipidemia in patients with bipolar disorder in clinical remission. J Affect Disord. 141(2):484–7.

245. Stemmle PG, Kenna HA, Wang PW, Hill SJ, Ketter TA, Rasgon NL. Insulin resistance and hyperlipidemia in women with bipolar disorder. J Psychiatr Res. 43(3):341–3.

246. Stenberg S. Psychosis and Blood Lipoids. Quantitative Variations of Total Cholesterin and Total Fatty Acids in the Blood. I. In Manic-Depressive Psychosis. Acta Med Scand. 1929;71(1):558–97.

247. Sundram S. 09-04 Plasma apolipoprotein E: roles and targets in schizophrenia and bipolar disorder. Acta Neuropsychiatr. 18(6):335.

248. Sussulini A, Pr, o A, Maretto DA, Poppi RJ, Tasic L, et al. Metabolic profiling of human blood serum from treated patients with bipolar disorder employing 1H NMR spectroscopy and chemometrics. Anal Chem. 81(23):9755–63.

249. Swardfager W, Hennebelle M, Yu D, Hammock BD, Levitt AJ, Hashimoto K, et al. Metabolic/inflammatory/vascular comorbidity in psychiatric disorders; soluble epoxide hydrolase (sEH) as a possible new target. Neurosci Biobehav Rev. 87:56–66.

250. Swartz CM. Albumin decrement in depression and cholesterol decrement in mania. J Affect Disord. 19(3):173–6.

251. Tatebayashi Y, Nihonmatsu-Kikuchi N, Hayashi Y, Yu X, Soma M, Ikeda K. Abnormal fatty acid composition in the frontopolar cortex of patients with affective disorders. Transl Psychiatry Psychiatry. 2:e204.

252. Theodoropoulou S, Gialouris AG. Lipids and mental disorders: Evidence, uncertainties and perspectives. Psychiatriki. 30(2):129–41.

253. Thomas EA, Dean B, Pavey G, Sutcliffe JG. Increased CNS levels of apolipoprotein D in schizophrenic and bipolar subjects: implications for the pathophysiology of psychiatric disorders. Proc Natl Acad Sci U S A. 98(7):4066–71.

254. Thomas EA, Copolov DL, Sutcliffe JG. From pharmacotherapy to pathophysiology: emerging mechanisms of apolipoprotein D in psychiatric disorders. Curr Mol Med. 3(5):408–18.

255. Thomas EA, Dean B, Scarr E, Copolov D, Sutcliffe JG. Differences in neuroanatomical sites of apoD elevation discriminate between schizophrenia and bipolar disorder. Mol Psychiatry. 8(2):167–75.

256. Tuncel OK, Sarisoy G, Bilgici B, Pazvantoglu O, Cetin E, Unverdi E, et al. Oxidative stress in bipolar and schizophrenia patients. Psychiatry Res. 228(3):688–94.

257. Tuncel OK, Sarisoy G, Bilgici B, Pazvantoglu O, Cetin E, Tuncel EK. Adipocytokines and ghrelin level of bipolar patients from manic episode to euthymic episode. Nord J Psychiatry. 72(2):150–6.

258. Turecki G, Grof P, Cavazzoni P, Duffy A, Grof E, Ahrens B, et al. Evidence for a role of phospholipase C-gamma1 in the pathogenesis of bipolar disorder. Mol Psychiatry. 1998;3(6):534–8.

259. Upton I. Ethyl-eicosapentaenoic acid in bipolar depression. Br J Psychiatry. 189(2):191.

260. Van Winkel R, Van Os J, Celic I, Van Eyck D, Wampers M, Scheen A, et al. Psychiatric diagnosis as an independent risk factor for metabolic disturbances: Results from a comprehensive, naturalistic screening program. J Clin Psychiatry. 69(8):1319–27.

261. Vargas HO, Nunes SO, Pizzo de Castro M, Bortolasci CC, Sabbatini Barbosa D, Kaminami Morimoto H, et al. Oxidative stress and lowered total antioxidant status are associated with a history of suicide attempts. J Affect Disord. 150(3):923–30.

262. Vemuri M, Kenna HA, Wang PW, Ketter TA, Rasgon NL. Gender-specific lipid profiles in patients with bipolar disorder. J Psychiatr Res. 45(8):1036–41.

263. Versace A, Andreazza AC, Young LT, Fournier JC, Almeida JR, Stiffler RS, et al. Elevated serum measures of lipid peroxidation and abnormal prefrontal white matter in euthymic bipolar adults: toward peripheral biomarkers of bipolar disorder. Mol Psychiatry. 19(2):200–8.

264. Vila-Rodriguez F, Honer WG, Innis SM, Wellington CL, Beasley CL. ApoE and cholesterol in schizophrenia and bipolar disorder: comparison of grey and white matter and relation with APOE genotype. J Psychiatry Neurosci. 36(1):47–55.

265. Vuksan-Cusa B, Marcinko D, Nad S, Jakovljevic M. Differences in cholesterol and metabolic syndrome between bipolar disorder men with and without suicide attempts. Prog Neuropsychopharmacol Biol Psychiatry. 33(1):109–12.

266. Wiener C, Rassier GT, Kaster MP, Jansen K, Pinheiro RT, Klamt F, et al. Gender-based differences in oxidative stress parameters do not underlie the differences in mood disorders susceptibility between sexes. Eur Psychiatry J Assoc Eur Psychiatr. 29(1):58–63.

267. Wollenhaupt-Aguiar B, Librenza-Garcia D, Bristot G, Przybylski L, Stertz L, Kubiachi Burque R, et al. Differential biomarker signatures in unipolar and bipolar depression: A machine learning approach. Aust N Z J Psychiatry. 54(4):393–401.

268. Woo HJ, Yu C, Kumar K, Reifman J. Large-scale interaction effects reveal missing heritability in schizophrenia, bipolar disorder and posttraumatic stress disorder. Transl Psychiatry Psychiatry. 7(4):e1089.

269. Wysokiński A, Strzelecki D, Kłoszewska I. Levels of triglycerides, cholesterol, LDL, HDL and glucose in patients with schizophrenia, unipolar depression and bipolar disorder. Diabetes Metab Syndr. 2015;9(3):168–76.

270. Yang J, Yan B, Zhao B, Fan Y, He X, Yang L, et al. Assessing the causal effects of human serum metabolites on 5 major psychiatric disorders. Schizophr Bull. 46(4):804–13.

271. Yao JK, Reddy RD. Metabolic investigation in psychiatric disorders. Mol Neurobiol. 2005;31(1):193–203.

272. Yui K, Kawasaki Y, Yamada H, Ogawa S. Oxidative Stress and Nitric Oxide in Autism Spectrum Disorder and Other Neuropsychiatric Disorders. CNS Neurol Disord Drug Targets. 2016;15(5):587–96.

273. Zak A, Slaby A, Tvrzicka E, Jachymova M, Macasek J, Vecka M, et al. Desaturases of fatty acids (FADS) and their physiological and clinical implication. Cas Lek Cesk. 2016;155(2):15–21.

274. Zaki N, Sadek H, Hewedi D, Hamed H, Raafat O. Metabolic profile and indices in a sample of drug-naive patients with schizophrenia and bipolar disorder. Middle East Curr Psychiatry. 2014;21(1):22–7.

275. Zeng J, Zhang Y, YuTao X, Liang S, Xue C, Zhang J, et al. Optimizing multi-domain hematologic biomarkers and clinical features for the differential diagnosis of unipolar depression and bipolar depression [Internet]. medRxiv; 2022 [cited 2022 Nov 30]. p. 2022.04.26.22274241. Available from: https://www.medrxiv.org/content/10.1101/2022.04.26.22274241v1
